# Selective adsorption of anionic and cationic dyes on mesoporous UiO-66 synthesized using a template-free sonochemistry method: kinetic, isotherm and thermodynamic studies†

**DOI:** 10.1039/d2ra06947d

**Published:** 2023-04-20

**Authors:** Alvin Romadhoni Putra Hidayat, Liyana Labiba Zulfa, Alvin Rahmad Widyanto, Romario Abdullah, Yuly Kusumawati, Ratna Ediati

**Affiliations:** a Department of Chemistry, Faculty of Science and Data Analytics, Institut Teknologi Sepuluh Nopember (ITS) Sukolilo Surabaya 60111 Indonesia rediati@chem.its.ac.id

## Abstract

In this study, template-free mesoporous UiO-66(U) has been successfully synthesized in shortened time by sonochemical methods and provided energy savings. The synthesized mesoporous UiO-66(U) demonstrated irregular morphology particle around 43.5 nm according to the SEM image. The N_2_ adsorption–desorption isotherm indicated an irregular, 8.88 nm pore width mesoporous structure. Ultrasonic irradiation waves greatly altered mesoporous materials. A mechanism for mesoporous UiO-66(U) formation has been proposed based on the present findings. Sonochemical-solvent heat saves 97% more energy than solvothermal. Mesoporous UiO-66(U) outperformed solvothermal-synthesized UiO-66(S) in adsorption. These studies exhibited that mesopores in UiO-66 promote dye molecule mass transfer (MO, CR, and MB). According to kinetics and adsorption isotherms, the pseudo-second-order kinetic and Langmuir isotherm models matched experimental results. Thermodynamic studies demonstrated that dye adsorption is spontaneous and exothermically governed by entropy, not enthalpy. Mesoporous UiO-66(U) also showed good anionic dye selectivity in mixed dye adsorption. Mesoporous UiO-66(U) may be regenerated four times while maintaining strong adsorption capability.

## Abstract 2

In this study, template-free mesoporous UiO-66(U) has been successfully synthesized in shortened time by sonochemical methods and provided energy savings.

## Graphical abstract

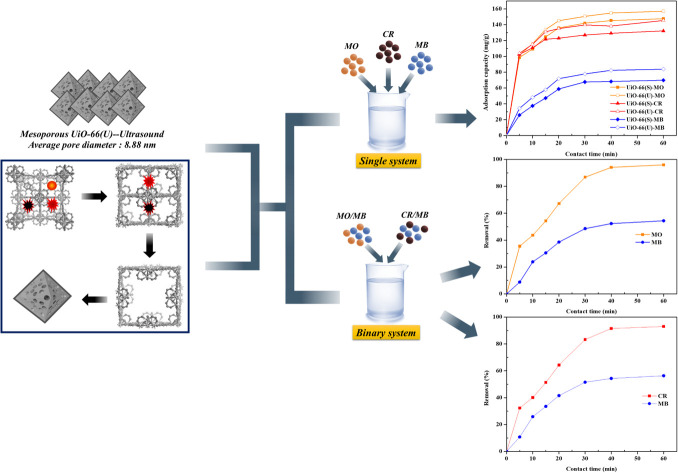

## Introduction

Water pollution could lead to several diseases, impair aquatic biodiversity, and inhibit the photosynthetic activity of aquatic plants.^1,2^ The massive growth of the textile industry is the leading cause of water pollution.^3^ Because of their vivid colors and fastness properties, dyes such as Congo Red (CR), Methylene Blue (MB), and Methyl Orange (MO) are often used in the textile industry.^4,5^ Common methods for removing dyes include membrane filtration, solar photo-oxidation, coagulation, ion exchange, and photocatalytic.^6–10^ However, these approaches have several drawbacks, including limited selectivity, low recovery, and significant operating and maintenance expenses. Adsorption is one of the most effective dye removal strategies because of its high efficiency, simple design and operation, low cost, and shortened processing time.^11,12^

A Metal Organic Framework (MOF) is a type of adsorbent that can remove the dye. Metal clusters coordinate with organic ligands to generate MOFs, which are crystalline hydride compounds.^13^ MOFs possess pore sizes and shapes that can be adjusted, a wide surface area, and a high affinity for different organic dyes.^14–16^ UiO-66, a MOF sub-class, is composed of a Zr metal cluster that binds with the H_2_BDC ligand (1,4 benzenedicarboxylic acid) to create a framework and has been extensively employed as a dye adsorbent.^17^ However, generally, UiO-66 is a microporous material and is often synthesized by solvothermal methods.^12,18–22^ Because dyes include numerous aromatic rings, the micropore structure can limit their diffusion and prevent them from interacting with active sites in the MOF structure.^11,23^ In comparison, the solvothermal method has many weaknesses, including long reaction times and requiring high temperatures and pressures.^24^ Recently, ultrasonication or sonochemical methods have been used to synthesize nanomaterials and develop mesoporous materials.^25,26^ The ultrasonication irradiation method is more environmentally friendly than the conventional method and gives a shorter reaction.^27^ Cavities caused by waves cause hot spots within the bubbles, which can help support MOF synthesis.^28^ In previous studies, the cavitation effect of ultrasound has been widely used in the preparation of mesoporous SiO_2_.^29–31^ So far, mesoporous MOFs have been synthesized with pre- or post-synthetic modifications, including surfactants as templates and defect formation or free templates with acids or bases.^32–34^ This method is unsuitable because the template-based synthesis method requires a post-synthetic process to remove the template, uses surfactants that are not environmentally friendly, and does not entirely remove the template.^34^ The approach to forming defects or free templates with low NaOH concentrations results in small and no mesopores.^33^ Ultrasound-assisted methods have been developed to synthesize mesoporous materials, including mesoporous hydroxyapatite,^26^ hierarchically porous NiO,^35^ and mesoporous ZnO, through sonochemical reactions without using templates or structural directing agents.^36^ Furthermore, the sonochemical method outperforms mechanical agitation in the formation of mesoporous and hierarchical microsphere structures.^36^ Abbasi *et al.*^37^ reported that the synthesis of Cu-BTC with ultrasound, in an average pore width according to BJH of 51.841 Å with an isotherm curve with a hysteresis loop; this result is much larger than the mechanochemical method with an isotherm curve without a hysteresis loop. To the best of our knowledge, there have been no investigations into the sonochemical synthesis of MOFs to produce mesoporous structures.

In this research, mesoporous UiO-66 was synthesized by the sonochemical method for dye adsorbent. The sonochemical was used to create mesoporous structures to increase adsorption performance. The selective adsorption of the anionic or cationic dyes is essential for developing the effectiveness of the adsorbent. Adsorption selectivity is influenced by three major factors: adsorbent and adsorbate size selectivity, ion exchange, and electrostatic attraction.^38^ MOFs are frequently anionic or cationic dye selective.^39–41^ This study aims to determine the adsorption performance of mesoporous UiO-66 on cationic and anionic dyes in single or binary solutions. As cationic and anionic dyes, Congo red (CR), methylene blue (MB), and methyl orange (MO) were chosen. In addition, a comprehensive study of adsorption isotherms and thermodynamics was also carried out.

## Experimental

### Materials

All materials utilized in this investigation were of analytical quality. C_8_H_6_O_4_ (benzene-1,4-dicarboxylic acid (H_2_BDC), 99%) was supplied by Sigma-Aldrich. C_3_H_7_NO (*N*,*N*-dimethylformamide (DMF), 99%), ZrCl_4_ (zirconium tetrachloride, 99%), CH_3_OH (methanol, 99%), C_14_H_14_N_3_NaO_3_S (methyl orange, 99%), C_16_H_18_ClN_3_S (methylene blue, 99%), and C_32_H_22_N_6_Na_2_O_6_S_2_ (Congo red, 99%) were purchased from Merck (see Table 1). In addition, demineralized water was purchased from a local market.

**Table 1:** Physicochemical properties of the selected dyes

Dye	Abbreviation	Chemical structure	Charge	*λ*_max_ (nm)
Methyl orange	MO	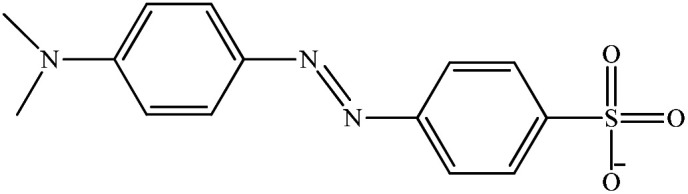	Anionic	464
Congo red	CR	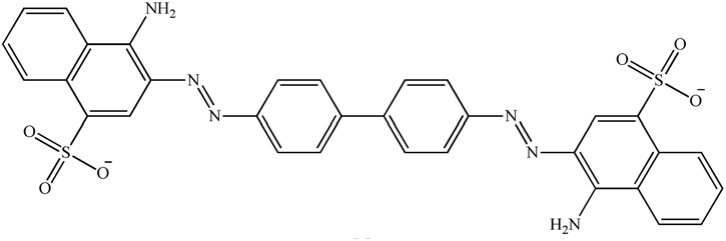	Anionic	498
Methylene blue	MB	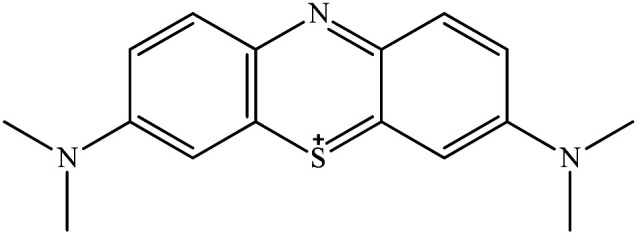	Cationic	665

### Methods

#### Conventional method for synthesis of UiO-66

**Fig. 1:**
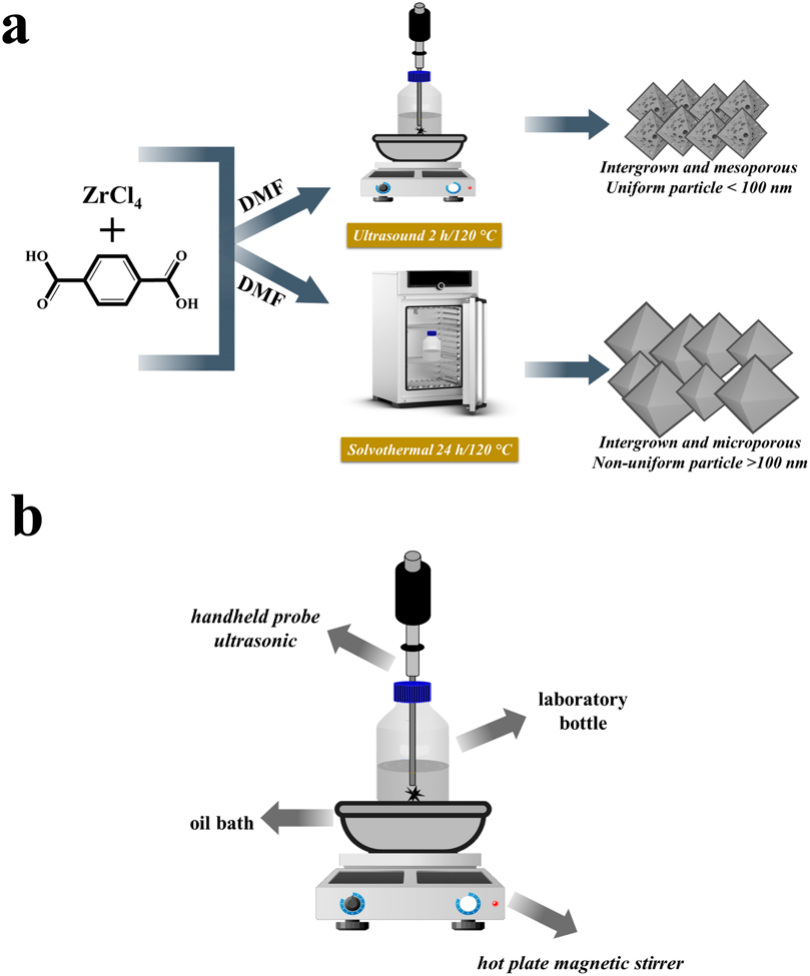
(a) Illustration of the preparation process of UiO-66(S) and mesoporous UiO-66(U); (b) set-up of ultrasonic reactor.

In the previous research, the solvothermal method was used to synthesize UiO-66 without ultrasonic irradiation, as seen in Fig. 1(a).^12^ Initially, 1.0487 g of (0.0045 mol) ZrCl_4_ precursor was added to 45 mL of DMF solution. In a separate bottle laboratory, 0.7483 g (0.0045 mol) of H_2_BDC precursor was dissolved in 45 mL of DMF solution using stirring for 15 minutes. The two solutions were then mixed and agitated for 30 minutes to obtain a final mixture. The resulting solution was then heated for 24 hours at 120 °C. Subsequently, the mixture was allowed to cool at room temperature overnight. 45 mL of DMF and 30 mL of methanol were used to rinse the product. Washing with 30 mL of methanol was carried out twice. The resulting precipitate was then dried in an oven at 90 °C for 3 hours. The synthesized material is denoted as UiO-66(S).

#### Ultrasound-assisted synthesis of mesoporous UiO-66

The sonochemical mesoporous UiO-66 synthesis technique modifies a previously described investigation.^42^ The first step was similar to the solvothermal technique in that the precursors, namely ZrCl_4_ and H_2_BDC, were dissolved in a DMF solution and agitated for 15 minutes. The two solutions were mixed and agitated in a laboratory bottle. As shown in Fig. 1(b), a reactor with a hot plate magnetic stirrer, astative, oil bath with a diameter of 20 cm and the sample is heated in a laboratory bottle with a speed of 60 °C h^−1^ from room temperature, and a handheld portable ultrasonic homogenizer probe (SONICA-2200 EP, 150 W, 30 kHz) was prepared. Subsequently, two hours of ultrasonication at 120 °C were done. The resulting mixture is cooled overnight at room temperature. The product was rinsed with DMF and methanol solutions after the solution was centrifuged to form a precipitate. The precipitate was then dried in an oven at 90 °C for 3 hours. The resulting material is denoted as mesoporous UiO-66(U).

#### Test point of zero charge (pH_zpc_)

The pH_zpc_ value is one of the factors used to calculate the surface charge of the UiO-66 adsorbent, which is affected by the pH of the solution. The pH_zpc_ experiment was conducted in the absence of dye in a 50 mL beaker that contained 20 mL of NaCl (0.1 M) solution. The approach of determining pH_zpc_ by adding salt (NaCl) is similar to previous studies.^43^ By utilizing 0.1 M HCl and 0.1 M NaOH, the starting pH of each beaker was changed from 1 to 12. Each beaker contained 0.1 g of adsorbent and was then wrapped in aluminum foil. Stirring was carried out for 24 hours at 100 rpm. Using a pH meter, the final pH of the solution was determined, and a point of zero charge (pH_zpc_) curve was produced by plotting both the original pH and ΔpH value (the discrepancy between the original and final pH). The line intersection of the resulting curve with the *x*-axis is the surface charge of the adsorbent (pH_zpc_) at pH = 0.^44^ Once the pH value is greater than pHzpc, the adsorbent's surface becomes positively charged. While the pH value is below pHzpc, the surface becomes negatively charged.^45,46^

#### Adsorption isotherms and kinetics in a batch experiment

The adsorption of CR, MB, and MO on the adsorbent in the single adsorption system was performed in a batch design. Prior to the adsorption process, the synthesized material was preheated at 90 °C for 3 hours for the activation process. Adsorption kinetics experiments were performed to identify the optimal contact period. Adsorption was carried out by adding 0.01 g of UiO-66 to a beaker that contained 20 mL of dye at concentrations of 100, 150, and 200 with varying contact times (5–60 minutes). After a specific time, the UiO-66 adsorbent was centrifuged from the dye solution for 10 minutes at 1500 rpm. By employing a UV-vis spectrophotometer, the concentration of the solution was observed at 464 nm, 665 nm, and 498 nm for MO, MB, and CR, respectively. The acquired data is displayed as a function of absorption capacity at a given time (*Q*_*t*_) and time in minutes (*t*).

The adsorption capacity and percentage of removal can be calculated by eqn (1) and (2), as follows:^47^1
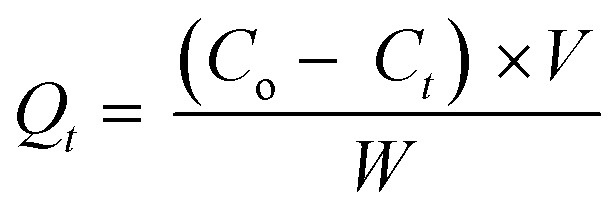
2
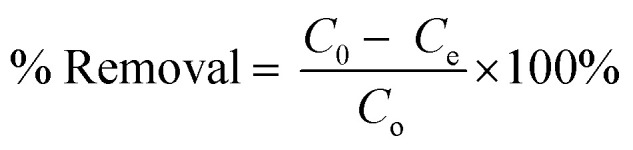
where *C*_o_ and *C*_*t*_ (mg L^−1^) are the initial and final concentrations of the adsorbate at equilibrium, respectively; *V* (L) is the volume of the adsorbate; and *W* (g) is the amount of adsorbent used. Adsorption was carried out in a beaker glass with a 10 mg/20 mL adsorbent dose at the optimum period, which was determined by altering the contact duration to investigate the adsorption isotherm. The concentration of the dye varied from 25 mg L^−1^ to 350 mg L^−1^. During the batch adsorption tests, the effectiveness of adsorption was also measured under different conditions, such as the pH of the solution, the amount of adsorbent, and the ionic strength.

#### Characterization

An XPert MPD diffractometer was used to record the X-ray diffraction (XRD) patterns of all synthesized materials. The diffractometer was set up with CuK (*λ* = 1.5406 Å) radiation, a voltage of 40 kV, and a current of 30 mA. At a wavenumber of 400–4000 cm^−1^, FT-IR spectra were obtained using an 8400S Shimadzu infrared spectrophotometer. SEM pictures of samples were taken using EDAX advanced microanalysis solutions. FESEM studied surface morphology (FE-SEM, JEOL JIB-4610F). N_2_ adsorption–desorption was studied using Quantachrome NovaWin Gas Sorption Analyzer. A PerkinElmer Pyris 1 analyzer conducted thermogravimetric analysis (TGA) on a 10 mg sample. Thermograms were taken at 10 °C min^−1^ air temperatures between 30 and 900 °C. Ultraviolet-visible (UV-vis) spectra recorded on a Thermo Scientific GENESIS 10S UV-vis Spectrophotometer for concentration of dyes after each adsorption.

#### Adsorption thermodynamic studies

The impact of temperature differences on the adsorption process was studied to evaluate the thermodynamics of adsorption and predict the dye adsorption mechanism. In addition, an analysis of thermodynamic factors was performed, including changes in Gibbs free energy, entropy, and enthalpy. Adsorption tests were performed at 30, 40, and 50 °C.

#### Performance of selective adsorption

To evaluate the degree of selectivity, 10 mg of adsorbent and 20 mL of a 20 mg L^−1^ dye mixture (10 mg L^−1^ in each dye) were mixed. Adsorption was carried out at 30 °C for 60 minutes at a pH of 7, while rotating at 350 rpm. Following the adsorption procedure, the adsorbent was separated, and UV-vis spectroscopy was employed to evaluate the concentration of dye residue in the supernatant. The competitive adsorption process for anionic and cationic dyes is evident from the selectivity values calculated from single adsorption isotherms using eqn (3) as follows:^44,48^3
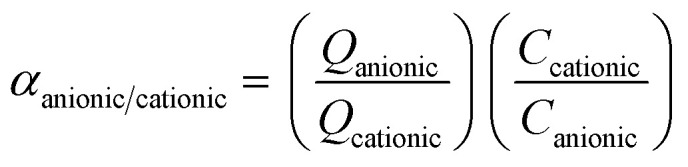
where *Q*_i_ and *C*_i_ (i: MO, CR or MB) represent the adsorption capacity at equilibrium and the concentration at equilibrium in the supernatant, respectively.

#### Regeneration performance evaluation

Desorption was done by stirring and centrifuging the spent adsorbent in a methanol solution at 2000 rpm for 90 minutes, three times, in order to determine the adsorbent's regenerative capacity. The desorption and regeneration studies were performed by mixing 10 mg of UiO-66 adsorbent with 100 mg L^−1^ of MO, CR, or MB solution. In the subsequent adsorption cycle, the recycled adsorbent is used.

## Results and discussion

### Synthesis of mesoporous UiO-66

**Fig. 2:**
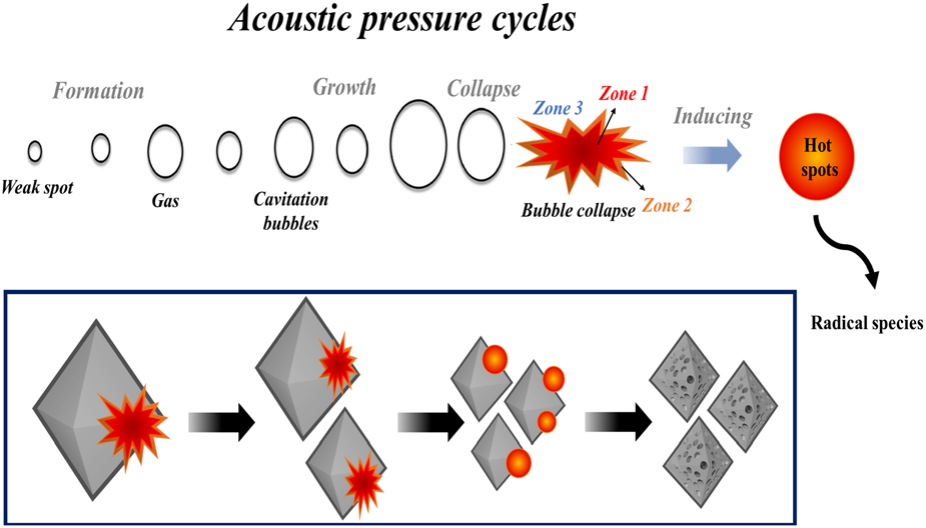
Illustration of cavitation-bubble collapse and sonofragmentation under ultrasonic conditions.

The synthesis of mesoporous UiO-66 with the ultrasonication-irradiation method provides a shorter reaction time. In contrast, the time used in the conventional method (solvothermal), which is 24 hours, can be reduced to only 2 hours. Cavitation caused by ultrasonic waves causes the formation of hot spots inside the bubbles, which can be useful for accelerating the synthesis of MOF.^28,49^ The presence of acoustic flow can lead to sonocrystallization and sonofragmentation (Fig. 2).^50^ There are four possible mechanisms for sonocrystallization and sonofragmentation, including collisions between particles, collisions of particles with ultrasonic probes, collisions with particle walls, and particle shock wave interactions. This phenomenon will result in the formation of nanoscale UiO-66 crystals, however the quantity of crystals will be mostly owing to the fragmentation of large crystals into nanoscale crystals.^50^ The yield produced by the ultrasonication irradiation method (97%) is higher than the yield produced by the stoichiometric solvothermal method (87%) (see in ESI†).

### XRD analysis of mesoporous UiO-66

**Fig. 3:**
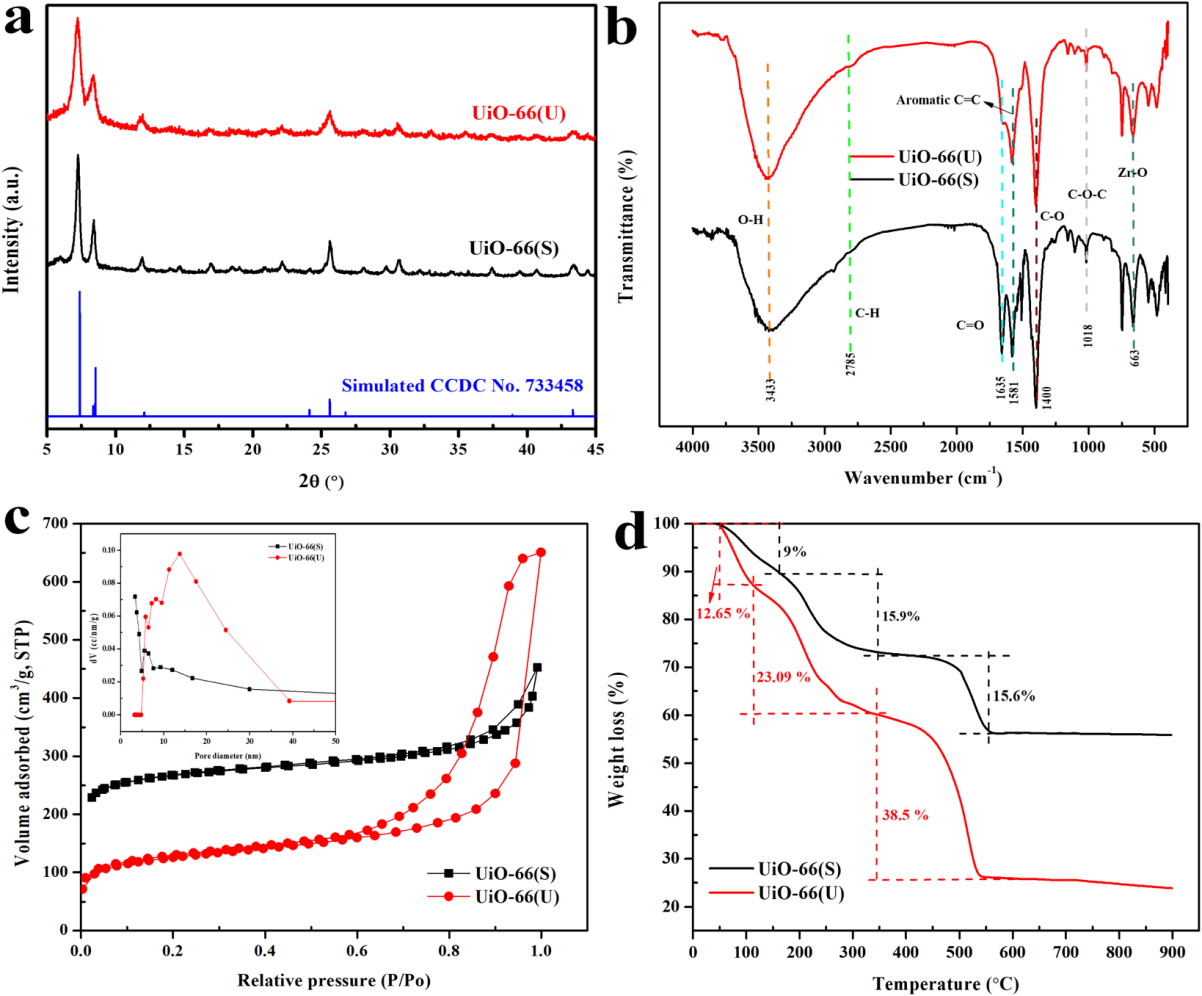
(a) XRD patterns, (b) FTIR spectra, (c) the nitrogen adsorption–desorption isotherms and BJH pore size distribution, (d) TGA curves of UiO-66(S) and mesoporous UiO-66(U).

Fig. 3(a) compares the physical properties of mesoporous UiO-66(U) with UiO-66(S). The peak position and intensity of the typical peaks of UiO-66 crystals are consistent with prior research.^51^ Table S1† provides information about the peak positions of the typical UiO-66 characteristics. The presence of three peaks in mesoporous UiO-66(U) and UiO-66(S) and the absence of any impurity peaks suggested that UiO-66 was properly synthesized.^52^ However, the XRD peak intensity of mesoporous UiO-66(U) synthesized *via* ultrasound was lower than that of UiO-66(S) synthesized *via* solvothermal. This phenomenon can be explained by the destruction of the UiO-66 framework due to ultrasonic energy, which significantly influences the collapse of part of the framework structure, leading to the formation of an irregular crystal structure.^36,53^ Therefore, UiO-66 treated with ultrasonic irradiation has a more open structure than conventionally synthesized UiO-66. Meanwhile, the expansion of XRD diffraction peaks can be associated with reduced crystal size or microstructure distortion due to the ultrasonic cavitation effect, which can provide an intensive mechanical shock resulting in bubble collapse.^35,54^

### FTIR analysis of mesoporous UiO-66

Fig. 3(b) presents the FTIR spectra of UiO-66 material synthesized by ultrasonication and solvothermal methods. Both mesoporous UiO-66(U) and UiO-66(S) materials exhibited absorption bands at the same wavenumber. The FTIR spectra of these materials revealed firm absorption peaks at 3300–3400 cm^−1^, which corresponded to the carboxylate's O–H stretching vibration and were partially due to water physisorption. At wave numbers of around 663 cm^−1^ and 1390–1400 cm^−1^, the existence of Zr–O stretching vibrations and C–O stretching vibrations from the carboxylic acid group validated the Zr coordination bond of the UiO-66 structure.^55^ The C=O vibrational absorption band of carboxylate appeared at a wavelength of 1630–1660 cm^−1^. In addition, absorption at 1580 cm^−1^ indicated the presence of aromatic C=C bonds in organic ligands derived from the benzene structure.^56^ According to the study done by Abid *et al.*,^15^ synthesized UiO-66 had an appropriate absorption peak.

### N_2_ adsorption–desorption analysis of mesoporous UiO-66

Fig. 3(c) depicts the N_2_ adsorption–desorption isotherm curves of mesoporous UiO-66(U) and UiO-66(S) materials. The UiO-66(S) adsorption isotherm synthesized by the solvothermal method exhibited a type I isotherm with a hysteresis loop of type H4 following IUPAC classification results in the presence of a small hysteresis.^57,58^ This type I curve demonstrates a characteristic of microporous materials in which the volume of gas adsorbed rises quickly at low relative pressures owing to adsorbed gas molecules interacting with portions of the solid surface to form a monolayer. The N_2_ adsorption–desorption isotherm curve was similar to previous studies on solvothermal synthesized UiO-66.^12,55,58^ The N_2_ adsorption–desorption isotherm curve of the mesoporous UiO-66(U) material shows a mixed type I and IV isotherm with a sharp increase in N_2_ adsorption at low pressures, followed by a constant rise until an H1 type hysteresis loop is found at high pressure.^48^ Adsorption also increased at *P*/*P*_0_ > 0.60 (mesoporous UiO-66(U)), indicating N_2_ adsorption at the mesoporous site. In addition, mesoporous UiO-66(U) material has a less specific surface area and micro-volume than UiO-66(S), namely 452.9 m^2^ g^−1^ and 0.1874 cm^3^ g^−1^ (Table 2). Due to the cavitation impact of ultrasonication, which could prevent crystallization and particle formation, the specific surface area of UiO-66 intra-particles decreases, resulting in reduced pores or cavities. However, there was an improvement in pore volume and mesopore diameter when compared to UiO-66(S), namely 0.9192 cm^3^ g^−1^ and 13.74 nm, respectively. The formation of this mesoporous material correlates with the SEM results, where a narrow gap between the particles is observed, which can be useful for accelerating the adsorbate diffusion during the adsorption process.^48^ These results are in accordance with the Abbasi *et al.*^37^ work, where the surface area of CuBTC produced by the ultrasonication method (U-CuBTC) (371 m^2^ g^−1^) is lower than that produced by the mechanical method (M-CuBTC) (1034 m^2^ g^−1^).^37^ In addition, the N_2_ adsorption isotherm types of M-CuBTC and U-CuBTC were types I and VI, respectively, and the MOF produced through ultrasonication formed mesopores with hysteresis loops. Because of the cavitation effect of the ultrasonication process, the mesoporous UiO-66(U) material exhibited the greatest mesoporous volume dispersion. It is supported by prior studies suggesting that the average pore diameter and mesopore diameter of U-CuBTC were greater than those of M-CuBTC (1.93 nm and 3.42 nm, respectively).^37^ On the other hand, an increase in the mesoporous volume and a decrease in the surface area of the material treated with ultrasonic irradiation can be associated with severe damage to the material.^59^ As shown in the XRD pattern of mesoporous UiO-66(U), the crystal structure of mesoporous UiO-66(U) is nearly destroyed owing to collapse. The UiO-66 micropore channel is therefore eliminated. Consequently, it can be deduced that ultrasonic irradiation plays a crucial role in resulting mesopores in a shorter amount of time and without the requirement for a template.

**Table 2:** Textural parameters of UiO-66(S) and mesoporous UiO-66(U)

Materials	BET surface area (m^2^ g^−1^)	Mesopore volume (cc g^−1^)	Mesopore diameter (nm)	Average pore diameter (nm)
UiO-66(S)	825.7	0.3031	3.396	3.39
Mesoporous UiO-66(U)	452.9	0.9192	13.74	8.88

### TGA analysis of mesoporous UiO-66

The material curves mesoporous UiO-66(U) and UiO-66(S) revealed three phases of sample weight loss throughout the heating process, as shown in Fig. 3(d). The thermal analysis curves of UiO-66(S) and mesoporous UiO-66(U) demonstrated a weight loss in the first stage, namely a 9% loss between 40 and 122 °C and a 12.65% decline between 40 and 107.5 °C, respectively. Due to the evaporation of the methanol solvent and physically adsorbed water that was still trapped in the UiO-66 framework or adsorbed on the surface of the nanoparticles, the sample's mass decreased. The weight decrease of the mesoporous UiO-66(U) material was more than that of the UiO-66(S) material, indicating greater water molecule adsorption.^4^ The second weight loss occurred at temperatures ranging from 140 to 280 °C (15.99%) for UiO-66(S) and 112 to 282 °C (23.09%) for mesoporous UiO-66(U). This was the decomposition stage of the solvent, unreacted organic ligands, and DMF trapped in the UiO-66 framework.^55^ The temperature range of 153–155 °C corresponds to the boiling point of DMF.^60^ The third weight loss was observed between 450 and 560 °C (15.60%) for UiO-66(S) material and between 284 and 560 °C (38.5%) for mesoporous UiO-66(U) material. This material lost weight owing to the dehydroxylation of two water molecules per cluster of [Zr_6_O_4_(OH)_4_]^12+^ into [Zr_6_O_6_]^12+^ clusters in UiO-66 crystals.^61,62^ Abid *et al.*^15^ show that ZrO_2_ is produced when the UiO-66 framework is damaged by the decomposition of the BDC ligand, which serves as the framework's linker. In the 30–900 °C temperature range, UiO-66 synthesized by ultrasonication and conventional methods lost about 74.24 and 40.59% of their weight, respectively. The high energy produced by the cavitation phenomena might enhance the unpredictability of the Brownian motion of the UiO-66 molecule, preventing the production of regular crystals and hence decreasing the thermal stability of the mesoporous UiO-66(U).^63^ The smaller crystal size and crystallinity of mesoporous UiO-66(U) compared to UiO-66(S) might also contribute to the higher weight reduction since thermal radiation would be transmitted more readily and effectively at lower temperatures, resulting in convective currents. A high heat load caused phase change and mass loss.^63,64^ In addition, mesoporous UiO-66(U) thermal stability was slightly decreased, which could be related to the missing linker defect caused by ultrasonic irradiation in the synthesis process.

### FESEM and SEM analysis of mesoporous UiO-66

**Fig. 4:**
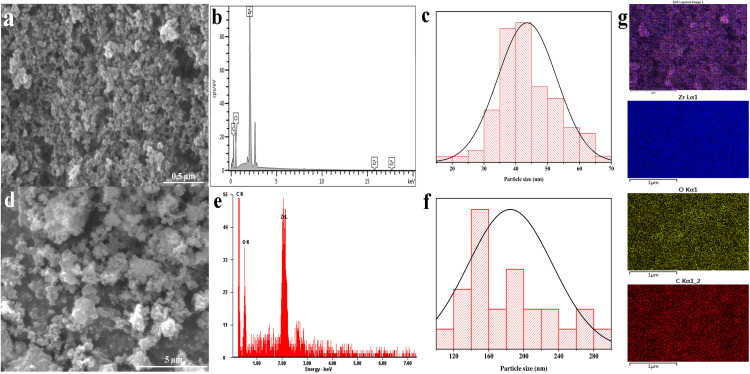
(a) EDT-FESEM image, (b) EDX spectra, (c) particles size distribution of mesoporous UiO-66(U), (d) EDT-SEM image, (e) EDX spectra, (f) particles size distribution of UiO-66(S), (g) elemental EDX mapping of mesoporous UiO-66(U).

Fig. 4 depicts the SEM and FESEM morphologies of the mesoporous UiO-66(U) and UiO-66(S) materials. The particle surface is agglomerate and irregular in the solvothermal synthesis of UiO-66(S) (Fig. 4(d)). A similar morphology was observed in the mesoporous UiO-66(U) material, where the particles intergrew and aggregated (Fig. 4(a)). These findings are similar to those of other studies.^4,55^ The solvothermal synthesis of UiO-66(S) yielded large agglomerated particles with an average diameter of 175 nm. This particle size is less than 200 nm compared to the UiO-66 reported earlier by Cavka *et al.*^51^ However, the surface of the mesoporous UiO-66(U) particles collapses, revealing fissures and holes in the UiO-66 structure. This is due to the incorporation of mesopores into the structure of UiO-66. The production of UiO-66 by ultrasonic irradiation in a precursor solution can result in high-speed particle collisions.^53^ Mesoporous UiO-66(U) particles were around 43.5 nm, which is smaller than UiO-66(S) particles. This is consistent with XRD results, which show that mesoporous UiO-66(U) crystallinity is lower than that of UiO-66(S). It should be noted that the decrease in crystallinity is related to the formation of small mesoporous UiO-66(U) nanoparticles. The cavitation effect induces more crystal nucleation than crystal growth, reducing in the average size of the mesoporous UiO-66(U) material.^65^ Moreover, Abbasi and Rizvandi compared sonochemical synthesis to conventional synthesis, with sonochemical synthesis or ultrasonication taking 60 minutes to create the 80 nm average particle size against 24 hours at 80 °C for regular synthesis to produce the 140 nm average particle size.^27^ Sargazi *et al.*^28^ also synthesized thorium MOF in an ultrasound bath at 40 °C for 21 minutes, yielding particles with an average size of 27 nm and uniform morphology. This approach outperforms the traditional process (85 °C, 60 minutes), in which the particles are not homogeneous and frequently agglomerate.^28^ The cavitation impact of ultrasonication can damage and break down particles. Cavitation might create collapsing microbubbles and huge amounts of local energy.^54,66^ The particle size of mesoporous UiO-66(U) is more uniform than that of UiO-66(S) according to the particle distribution curve (Fig. 4(c) and (f)), which is consistent with previous studies.^67–69^ Furthermore, there is a small gap between the particles, which agrees with the BET data showing that mesoporous UiO-66(U) contains more and bigger mesopores than UiO-66(S). EDX spectra are used to identify the material composition and element distribution in the resultant material, as exhibited in (Fig. 4(b) and (e)). The EDX values for UiO-66(S) and mesoporous UiO-66(U) are shown in Table S2.† The EDX spectrum data demonstrates that the UiO-66 substance is composed of carbon (C), oxygen (O), and zirconium (Zr). Both materials have almost the same quantity of elemental oxygen. However, the proportions of elements C and Zr differ, with element C in mesoporous UiO-66(U) decreasing due to ultrasonication. Fig. 4(g) displays the elemental distribution of the mesoporous UiO-66(U) material composition as determined by EDX mapping.

### Possible mechanism for formation of mesoporous UiO-66 *via* sonochemical reaction

**Fig. 5:**
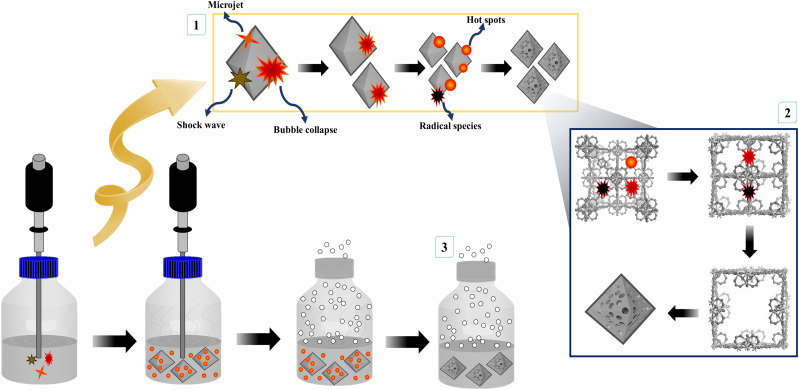
(a) XRD patterns, (b) FTIR spectra, (c) the nitrogen adsorption–desorption isotherms and BJH pore size distribution, (d) TGA curves of UiO-66(S) and mesoporous UiO-66(U).

It is well known that high-intensity ultrasonic irradiation can cause cavitation and heating (hot spots), which might lead in processes including hydrolysis, redox, and dissolution.^36^ The heating effect of ultrasonication and the strong ultrasonic cavitation effect can produce many bubbles in the precursor solution (Fig. 5). This effect can promote crystal growth in a short time. The sonohydrolysis of the UiO-66 precursor solution at the interfacial area on the interior of the collapsing bubble, where high temperatures and pressures are produced, results in the pyrolysis of water into H^+^ and OH^−^ radicals.^70^ The low vapor pressure and low viscosity of DMF favor the acoustic cavitation process, and the presence of water in the solution increases the hydrolysis rate of the UiO-66 precursor on the surface of the collapsed bubble.^36,71^ In this case, the high and prolonged ultrasonic irradiation power can lead to the formation of ordered particles, the collapse of the cavitation bubble, and the hydrolysis of the UiO-66 precursor on the bubble surface. Therefore, the mesoporous UiO-66(U) particles are small with a uniform size, where the particles are formed according to the shape and size of the collapsed bubble (inter-bubble space). High energy and highly irregular collapsing bubbles form mesoporous UiO-66(U) particles with larger and uneven pores.^71,72^ Aside from the causes above, the holes left on the interior or surface of the particles can be generated by the system rapidly cooling, forcing the gas to quickly escape from the precursor solution, producing a mesoporous structure. With changes in external pressure and temperature, the holes inside the particles are linked together to form irregular holes.^26^ UiO-66 is a form of MOF with unsaturated metal sites/open-metal sites where many labile ligands permit BDC linker damage owing to the sonolysis process, as shown by the reduction in the proportion of C atoms in the EDX characterization of the mesoporous UiO-66(U) structure.^73^ It should be noted that chemical bonds can be broken through intensive heat or hot spots generated by acoustic cavitation, resulting in the removal of organic linkers and enlargement of the MOF cavity. The breaking of chemical bonds in sonolysis can be triggered by intense heat within the framework.^74^ Sonolysis-induced damage to the framework may lead to the loss of organic linkers, resulting in partly disintegrated MOFs with mesoporous structures.^75,76^

### Adsorption performance studies

#### Effect of adsorbent dosage on the adsorption of dyes

**Fig. 6:**
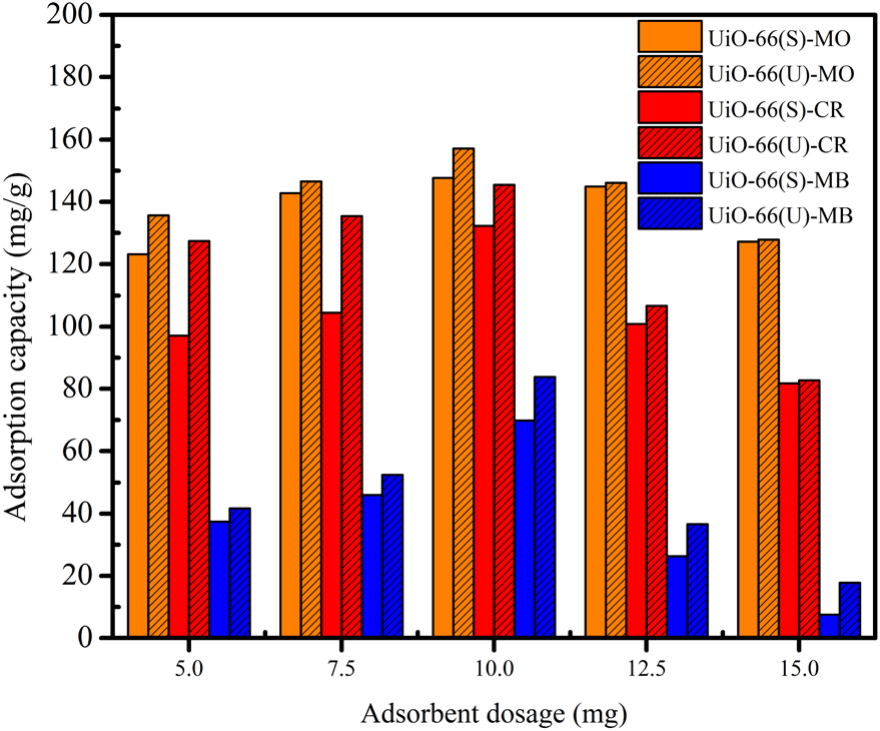
Effect of dosage on the adsorption on the adsorption MO, CR and MB on UiO-66(S) and mesoporous UiO-66(U).

The impact of various UiO-66 adsorbent dosages on dye adsorption was studied using a constant concentration and volume of dye solution at varying UiO-66 masses, as exhibited in Fig. 6. The adsorption capacity of UiO-66 for the three dyes was enhanced when the adsorbent dosage was raised from 5 to 10 mg. Increasing the adsorbent dosage from 10 to 15 mg might reduce in adsorption capacity. The highest adsorption capacity was achieved at 10 mg of adsorbent, which might be attributed to various factors. At this dosage, the chance of adsorbent surface collisions with dye molecules is substantially more extensive, and there are more adsorption sites for dye adsorption.^44,77^ Excessive adsorbent addition (from 10 to 15 mg) may increase the aggregation effect due to the presence of more active sites, thereby decreasing the adsorption surface sites.^78,79^ Consequently, 10 mg was the optimal dose for all subsequent adsorption studies.

### Study of the effects of contact time and kinetics

The influence of contact time between adsorbent and adsorbate is an essential parameter in practical adsorption applications. The influence of contact duration on dye adsorption was examined using dye concentrations (MO, CR, and MB) of 100, 150, and 200 mg L^−1^, 10 mg adsorbent dosage, 20 mL solution volume, and a solution pH of 7. The adsorption capacity of the three dyes improved. Because the dye molecules do not entirely fill many adsorption sites, the time decreases dramatically from 0 to 15 minutes (Fig. S1 and S2†). However, after 40 to 60 minutes, the adsorption rate tends to slow down owing to a drop in the number of sites for adsorption, resulting in a reduction in adsorption capacity, indicating a condition of equilibrium or saturation. Furthermore, an enhancement in the repulsion between the dye molecules and the adsorbent might be one of the other variables causing a minor increase in adsorption capacity as contact time increases.^5,13^ In the succeeding experiment, 60 minutes were confirmed to be the ideal duration to reach equilibrium.

The MO and CR (anionic) adsorption capacities on UiO-66 were significantly greater than those of MB (cationic). The MO adsorption capacities of the mesoporous UiO-66(U) and UiO-66(S) samples at 30 °C and 100 mg L^−1^ were 157.12 and 147.7 mg g^−1^, respectively. At 60 minutes, the MB adsorption capacities of mesoporous UiO-66(U) and UiO-66(S) were 69.8 and 83.9 mg g^−1^, respectively. Furthermore, the adsorption capacity of CR at the start of adsorption is larger than the adsorption capacity of MO. This might happen because CR dyes have more electronegative sites than MO dyes, which makes it easier for them to stick to the positively charged UiO-66 surface.^13,80^ The advantage of this CR adsorption capacity did not last long since, with increasing contact time, the adsorption capacity of CR was less than the adsorption capacity of MO because CR dye has a bigger size and molecular weight than MO dye. Therefore, after the active site on the outer surface is saturated, the dye adsorption process will occur in the interior pores through diffusion. This process might benefit smaller dye molecules more gradually adsorption than the external adsorption process does faster for bigger dye molecules.^81^ It was feasible because the ultrasonication approach could increase the volume and pore diameter of the material owing to the cavitation effect, generating crystal defects in the MOF structure.^37,54^ The pore size distribution of mesoporous UiO-66(U) material was substantially more considerable than that of UiO-66(S), indicating that a larger pore volume induces an increase in adsorption activity. Furthermore, the crystallinity or irregularity of the mesoporous UiO-66(U) material framework will provide an adsorption process with more active sites.^56^ Therefore, more opening pores are available, facilitating the diffusion of the dye molecules in the UiO-66 structure. Thus, the mesoporous MOF design is a valuable target for enhancing adsorption capacity.

Adsorption kinetics analysis was performed to investigate and comprehend the adsorption process and rate. Adsorption kinetics generic models include pseudo-first-order, pseudo-second-order, intraparticle diffusion, and Elovich models.^5,12,82^Eqn (4) expresses the pseudo-first-order adsorption kinetics equation as follows:

4 ln(*Q*_e_ − *Q*_*t*_) = ln *Q*_e_ − *K*_1_*t*

where *K*_1_ (1/min) denotes a pseudo-first-order rate constant. *Q*_e_ and *Q*_*t*_ (mg g^−1^) are adsorption capacities at saturation time and a certain time. Moreover, eqn (5) provides the quasi-second-order adsorption kinetics, as follows:^83^5
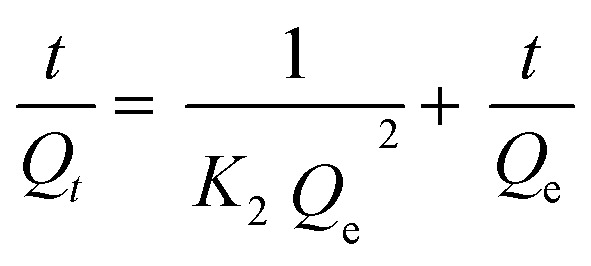
*K*_2_ (g mg^−1^ min^−1^) denotes a pseudo-second-order rate constant. *Q*_e_ and *Q*_*t*_ are the same as in eqn (4).

**Fig. 7:**
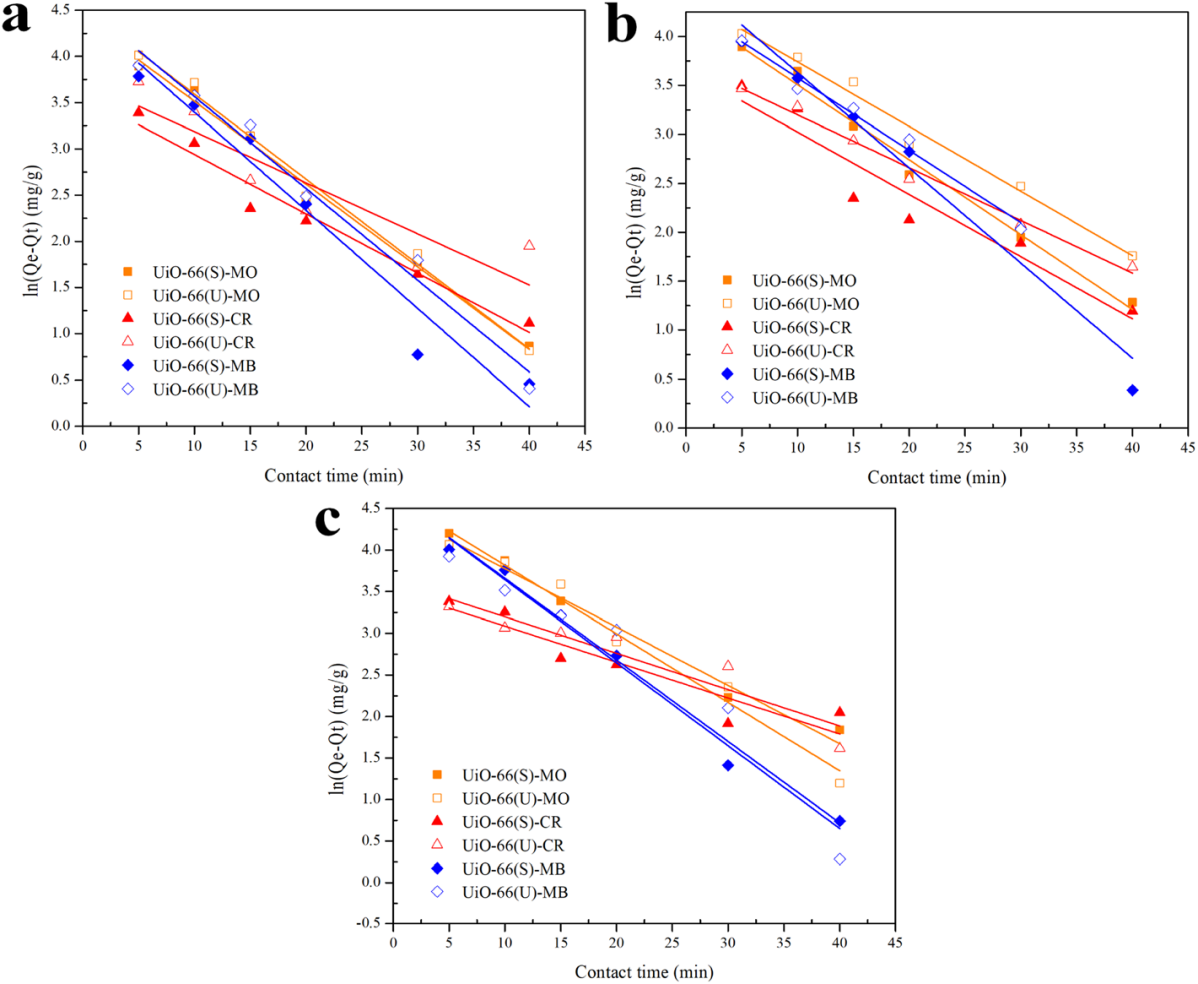
Model fits of the experimental data with pseudo-first-order model for adsorption of MO, CR and MB onto adsorbents at 30 °C and initial concentration of (a) 100, (b) 150, (c) 200 mg L^−1^.

**Fig. 8:**
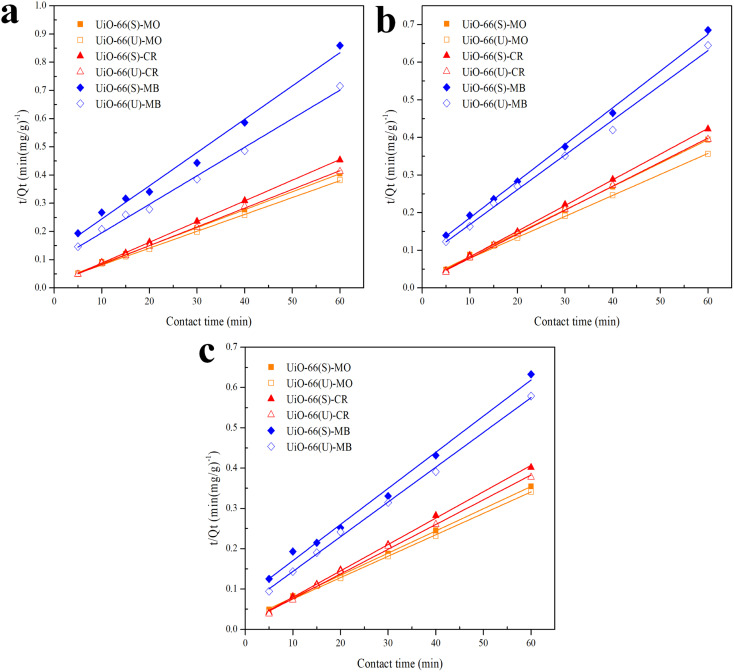
Model fits of the experimental data with pseudo-second-order model for adsorption of MO, CR and MB onto adsorbents at 30 °C and initial concentration of (a) 100, (b) 150, (c) 200 mg L^−1^.

**Fig. 9:**
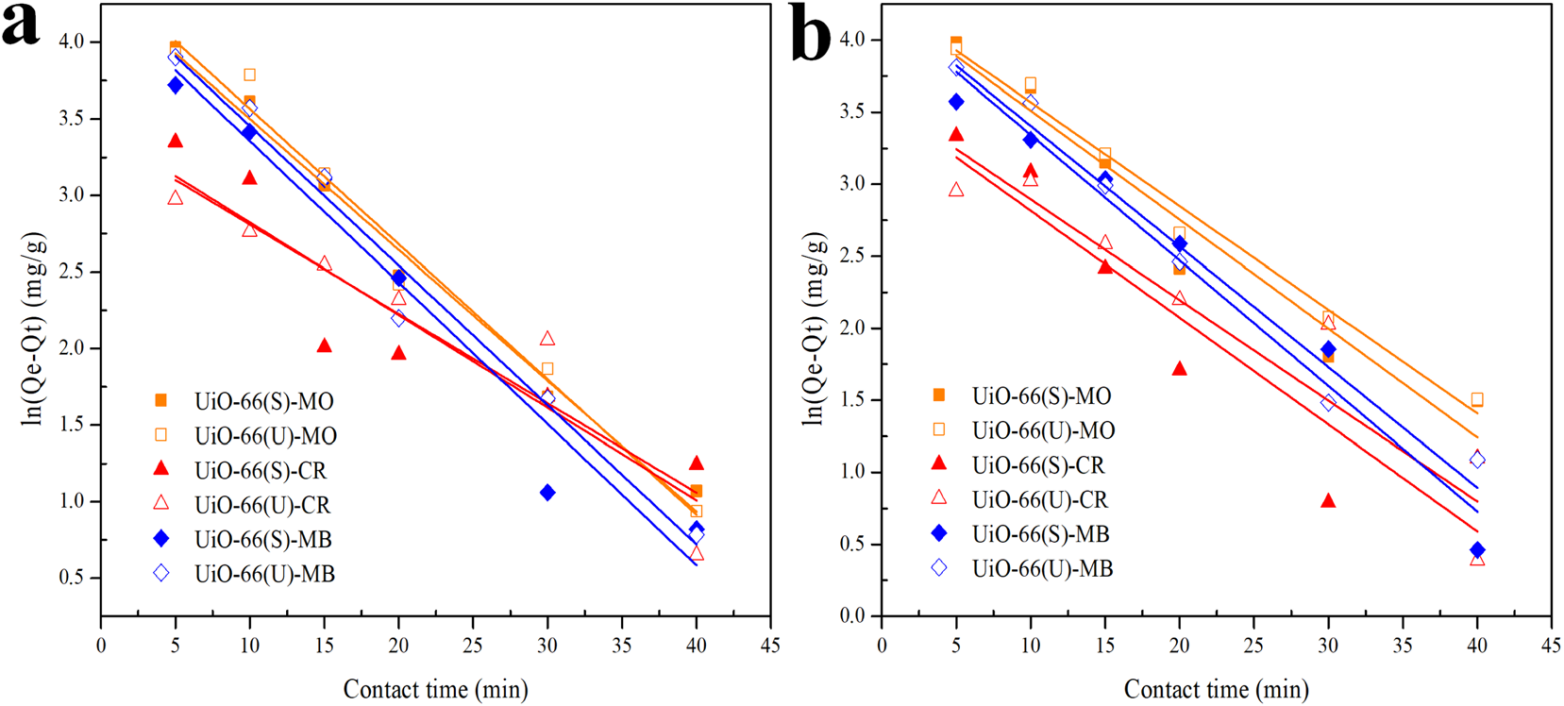
Model fits of the experimental data with pseudo-first-order model for adsorption of MO, CR and MB onto adsorbents at (a) 40 and (b) 50 °C.

**Fig. 10:**
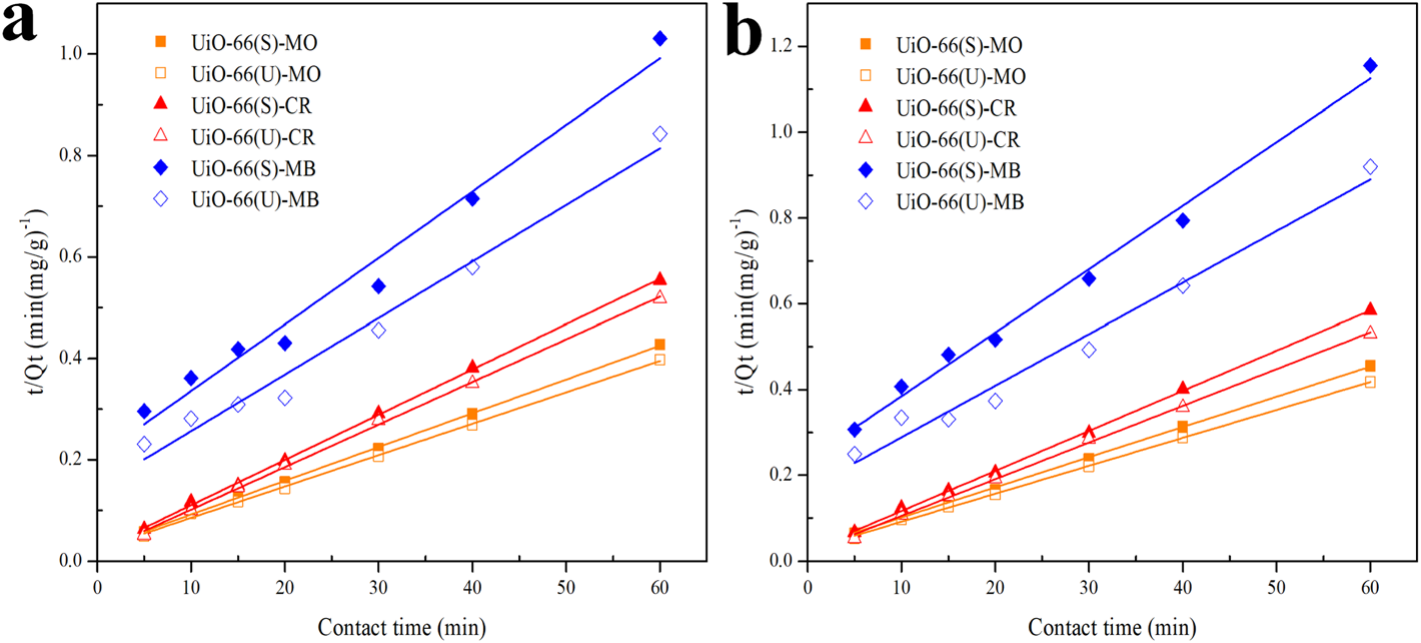
Model fits of the experimental data with pseudo-second-order model for adsorption of MO, CR and MB onto adsorbents at (a) 40 and (b) 50 °C.

The correlation coefficient value (*R*^2^) (Tables 3 and 4) for the pseudo-second-order kinetic model is near one and slightly higher than the other models based on fitting data (Fig. 7–10) for the two adsorption kinetics models. The pseudo-first-order kinetic model represents the physisorption process, while the pseudo-second-order kinetic model describes the chemisorption process.^4,77^ The pseudo-second-order adsorption kinetics model is more appropriate than the pseudo-first-order model for the adsorption of the three kinds of dyes. These results demonstrate that MO, CR, and MB adsorption happens by chemisorption, such as electron exchange between the adsorbent and the adsorbate.^44,77^ Additionally, the pseudo-second-order model's calculated *Q*_e_ value (*Q*_e, cal_) is closer to the experimental *Q*_e_ value (*Q*_e, exp_) than the pseudo-first-order model's calculated *Q*_e_ value (*Q*_e, cal_). It can also be shown that at higher temperatures, the adsorption capacity was reduced at high concentrations, suggesting that higher temperatures considerably slowed the adsorption rate. According to Table 4, the value of *K*_2_ increased as the temperature increased. However, when the initial concentration of the dye rises, the value of *K*_2_ at a constant temperature tends to decrease.^79,84^ Furthermore, the *K*_2_ value of most UiO-66(S) was found to be greater than the *K*_2_ value of mesoporous UiO-66(U) in all temperature change studies. This might be due to a change in the synthesis procedure that modifies the pore properties and surface charge of UiO-66, allowing it to attract more adsorbates.^85^

**Table 3:** Parameters of adsorption kinetic models with the variation of dyes initial concentration

Adsorbents	Dye	Co (mg L^−1^)	Pseudo-first-order model			Pseudo-second-order model			*Q*_e, exp_ (mg g^−1^)
			*Q*_e_ (mg g^−1^)	*K*_1_ (min^−1^)	*R* ^2^	*Q*_e_ (mg g^−1^)	*K*_2_ (g mg^−1^ min)	*R* ^2^	
UiO-66(S)	MO	100	64.367	0.0747	0.9718	156.250	0.00164	0.9993	147.694
		150	66.062	0.0716	0.9927	161.290	0.00152	0.9996	152.207
		200	94.179	0.0743	0.9911	181.818	0.00128	0.9996	169.369
	CR	100	33.535	0.0600	0.9828	136.986	0.00365	0.9998	132.274
		150	37.285	0.0612	0.9642	147.059	0.00328	0.9996	142.008
		200	42.640	0.0575	0.9356	153.846	0.00300	0.9989	149.364
	MB	100	53.069	0.0766	0.8906	84.7460	0.00084	0.9892	69.8390
		150	72.959	0.0782	0.9452	102.041	0.00109	0.9981	87.5710
		200	73.560	0.0789	0.9525	111.111	0.00104	0.9944	94.8430

Mesoporous UiO-66(U)	MO	100	70.492	0.0764	0.9706	166.667	0.00161	0.9994	157.127
		150	91.131	0.0728	0.9797	178.571	0.00138	0.9989	168.543
		200	95.919	0.0777	0.9885	188.679	0.00129	0.9987	175.830
	CR	100	46.810	0.0618	0.9309	151.515	0.00253	0.9995	145.451
		150	47.756	0.0617	0.9974	156.250	0.00273	0.9994	151.909
		200	48.965	0.0593	0.9340	163.934	0.00258	0.9978	158.917
	MB	100	66.887	0.0777	0.9443	99.010	0.00086	0.9948	83.8570
		150	74.918	0.0808	0.9024	108.696	0.00112	0.9943	93.0750
		200	75.528	0.0790	0.9287	116.279	0.00130	0.9980	103.655

**Table 4:** Parameters of adsorption kinetic models with the variation of temperature

Adsorbents	Dye	*T* (°C)	Pseudo-first-order model			Pseudo-second-order model			*Q*_e, exp_ (mg g^−1^)
			*Q*_e_ (mg g^−1^)	*K*_1_ (min^−1^)	*R* ^2^	*Q*_e_ (mg g^−1^)	*K*_2_ (g mg^−1^ min)	*R* ^2^	
UiO-66(S)	MO	30	64.367	0.0747	0.9718	156.250	0.00164	0.9993	147.694
		40	63.931	0.0735	0.9781	149.254	0.00184	0.9995	140.460
		50	66.500	0.0714	0.9763	140.845	0.00219	0.9990	131.786
	CR	30	33.535	0.0600	0.9828	136.986	0.00365	0.9998	132.274
		40	28.951	0.0567	0.9326	112.360	0.00384	0.9994	108.229
		50	27.947	0.0604	0.8861	106.383	0.00385	0.9994	102.681
	MB	30	53.069	0.0766	0.8906	84.7460	0.00084	0.9892	69.8390
		40	52.342	0.0729	0.9336	76.3360	0.00093	0.9801	58.2030
		50	52.248	0.0715	0.9541	67.568	0.00110	0.9921	51.9450

Mesoporous UiO-66(U)	MO	30	70.492	0.0764	0.9706	166.667	0.00161	0.9994	157.127
		40	68.862	0.0752	0.9717	161.290	0.00166	0.9986	151.109
		50	72.226	0.0715	0.9944	153.846	0.00170	0.9994	143.586
	CR	30	46.810	0.0618	0.9309	151.515	0.00253	0.9995	145.451
		40	29.303	0.0574	0.9515	119.048	0.00381	0.9990	115.826
		50	31.224	0.0606	0.9240	116.279	0.00410	0.9989	113.025
	MB	30	66.887	0.0777	0.9443	99.0100	0.00086	0.9948	83.8570
		40	59.394	0.0741	0.9568	89.2860	0.00087	0.9815	71.1420
		50	56.969	0.0716	0.9714	83.3330	0.00109	0.9807	65.2430

Using eqn (6), a kinetic data analysis of the intraparticle diffusion model could be used to figure out the rate of the control steps involved in the dye molecules getting into the adsorbent:^86^

6 *Q*_*t*_ = *k*_id_*t*^1/2^ + *C*

where *K*_id_ (mg g^−1^ min^0.5^) exhibits the intraparticle diffusion rate constant determined by the slope of *Q*_*t*_*vs. t*^0.5^, while *C* (mg g^−1^) presents the boundary layer thickness determined by the intercept value.^87^

**Fig. 11:**
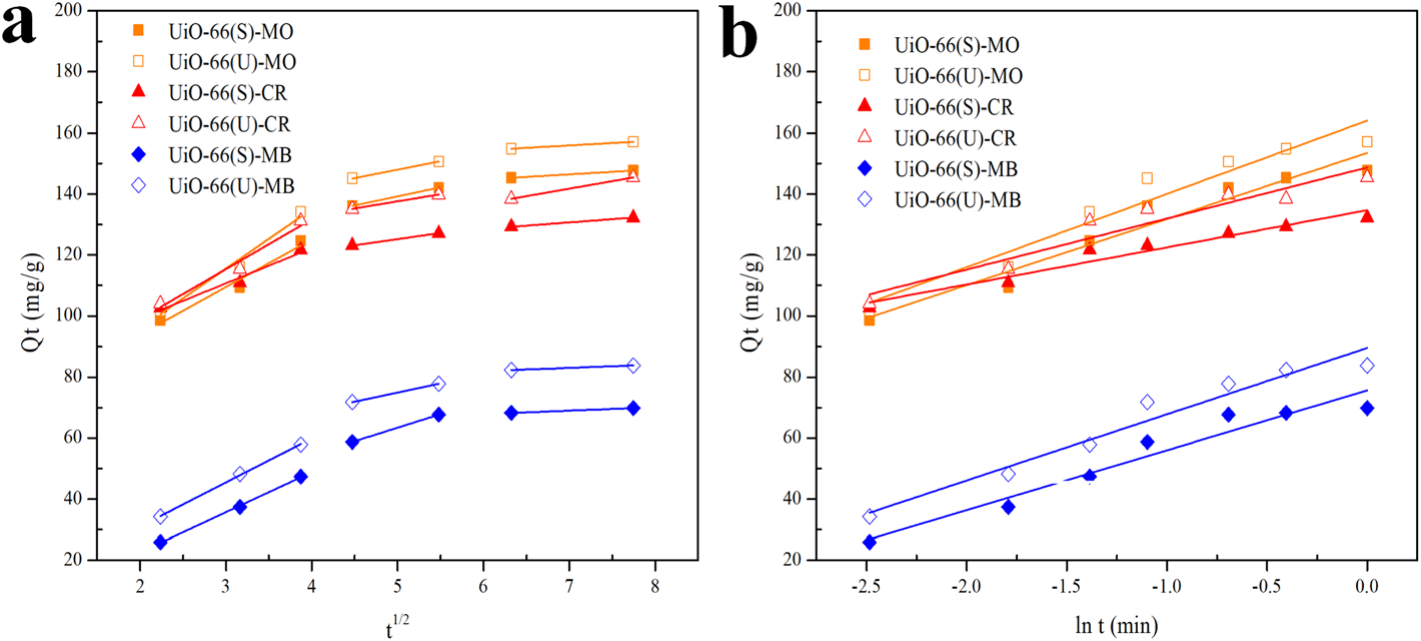
Model fits of the experimental data with (a) intraparticle diffusion model and (b) Elovich for the adsorption of MO, CR and MB onto synthesized materials at 30 °C and initial concentration of 100 mg L^−1^.

The intraparticle diffusion model plot is shown in Fig. 11(a). The three stages of the adsorption process are shown in the figure. The existence of external surface diffusion is evident in the first stage, characterized by a faster rise in the adsorption rate.^44^ The second step is decreased adsorption through intraparticle diffusion, with a significant drop in slope. The third step indicates that the adsorbate penetrates the UiO-66 pores and covers all the adsorbent surface's active sites.^44,88^Table 5 displays the values of *K*_id_ and *R*^2^. Another kinetic model, Elovich, depicts the chemisorption process and the heterogeneous adsorbent surface, hence modeling the many dye adsorption sites (Fig. 11(b)).^89^ Elovich model is stated in eqn (7):7
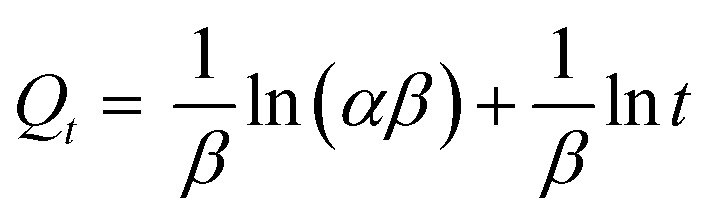
where the Elovich model parameters are *β* (g mg^−1^) and *α* (mg g^−1^ h^−1^). Table 5 displays the Elovich model parameter values. The characteristics of the Elovich model indicate the presence of a heterogeneous site distribution defined by varied chemisorption activation energies.^90^ The *R*^2^ value of the Elovich model was higher than that of the intraparticle diffusion model, suggesting that it was most appropriate.

**Table 5:** Parameters of intraparticle diffusion and Elovich model at 30 °C and initial concentration of 100 mg L^−1^

Adsorbent	Dye	Intraparticle diffusion			Elovich		
		*k*_id_ (g mg^−1^ min^1/2^)	*C* (mg g^−1^)	*R* ^2^	*α* (g mg^−1^ h^−1^)	*β* (g mg^−1^)	*R* ^2^
UiO-66(S)	MO	9.251	82.128	0.8583	25 511.903	0.0461	0.9453
	CR	5.180	96.352	0.8585	759 408.49	0.0820	0.9523
	MB	13.813	13.813	0.8564	924.38070	0.0509	0.9411

Mesoporous UiO-66(U)	MO	13.890	73.149	0.8399	22 022.091	0.0416	0.9414
	CR	7.4850	93.215	0.8736	83 298.749	0.0570	0.9595
	MB	20.963	20.963	0.8729	1335.9876	0.0460	0.9562

### Influence of initial dye concentration and isotherm analysis

**Fig. 12:**
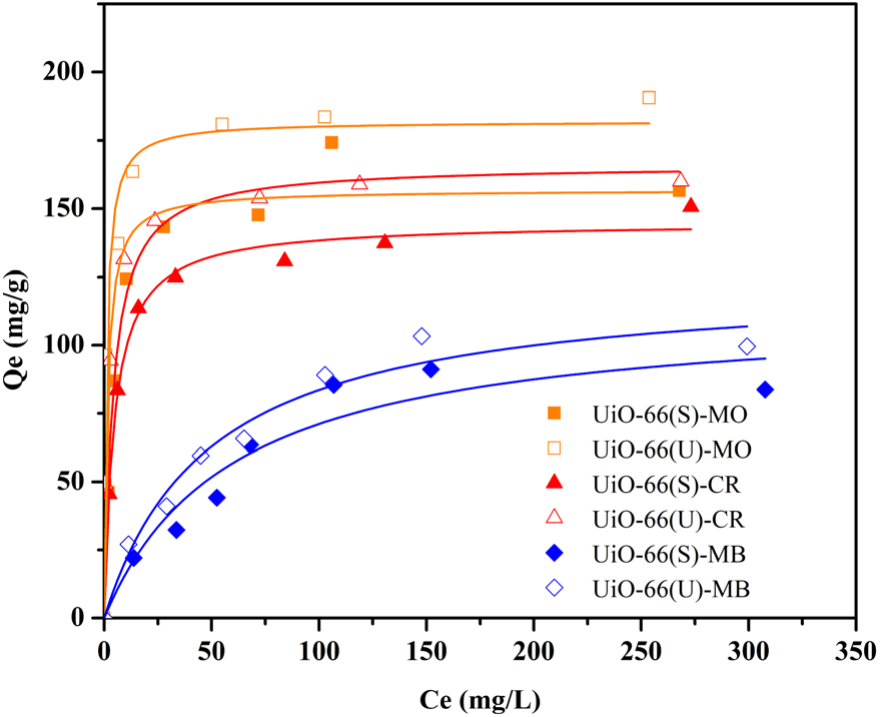
Effect of initial concentration of MO, CR and MB on the adsorption by UiO-66(S) and mesoporous UiO-66(U).

The subsequent adsorption studies were performed with changes in the starting concentration of the dye under equilibrium circumstances caused by contact time variations. The initial concentrations of the three dyes ranged from 25 to 350 mg L^−1^. As illustrated in Fig. 12, the three dyes' adsorption capacity improved with the higher dye solution's concentration. Moreover, adsorption capacity enhanced as the number of dye molecules adsorbed onto the adsorbent's surface increased. This demonstrates that raising the adsorbate solution concentration provides a driving force for the adsorbate to be adsorbed.^13,48^ The adsorption capacity values became consistent at a concentration of 300 mg L^−1^ due to the adsorbent being saturated by the adsorbate at that concentration. The mesoporous UiO-66(U) adsorption capability for the three dyes was greater than that of UiO-66(S). As indicated in Table 2, the BET surface area of mesoporous UiO-66(U) is less than that of UiO-66(S), although the MO, CR, and MB adsorption capacities are greater than those of UiO-66(S). It suggests that the mesoporous structure facilitates entry into the adsorption site and allows for a shorter diffusion route. Furthermore, mesopores can alleviate space constraints while increasing mass transfer.^19^ However, the MB adsorption capacity in both samples was pretty low. The positive surface charge of both samples causes this tendency, as shown by the point of zero charges (pH_zpc_) test.

**Fig. 13:**
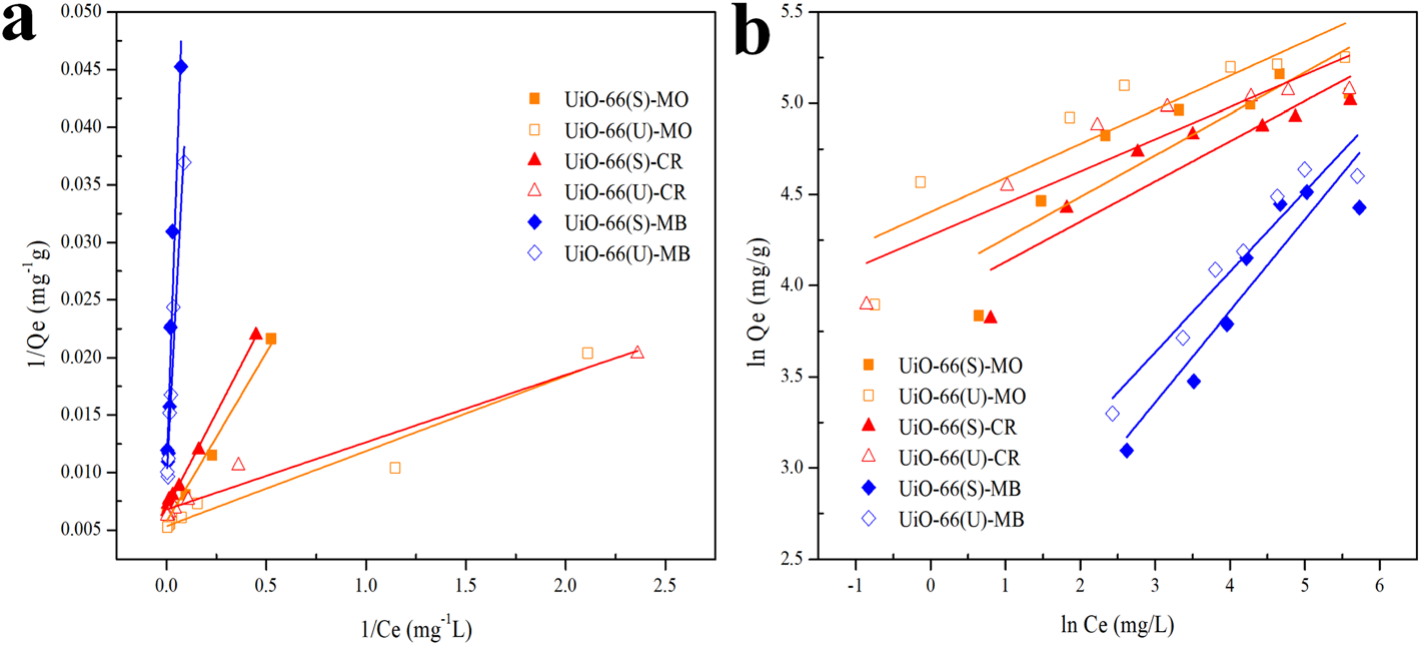
Model fits of (a) Langmuir and (b) Freundlich isotherms with experimental adsorption data of MO, CR and MB on UiO-66(S) and mesoporous UiO-66(U).

Isotherms are essential to determining adsorption efficiency and understanding adsorption mechanisms. Langmuir, Dubinin–Radushkevich (D–R), Freundlich, Scatchard, and Temkin adsorption isotherm models are extensively employed in isotherm studies. The Freundlich adsorption isotherm is an empirical function that indicates that the surface of the adsorbent is not homogeneous, that adsorption takes place in multilayers, and that adsorption capacity rises with increasing concentration.^77^ The Freundlich isotherm can be written as (eqn (8)):8
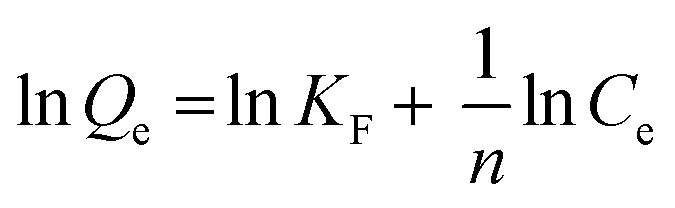
whereas *Q*_e_ (mg g^−1^) represents the adsorptive capacity throughout the process, *K*_F_ (mg g^−1^ (L mg^−1^)^1/*n*^) denotes the constant Freundlich, and 1/*n* exhibits the energy distribution factor at the adsorption site. Fig. 13(b) depicts plots of Freundlich adsorption isotherms by mesoporous UiO-66(U) and UiO-66(S) for MO, CR, and MB at 30 °C. Although the values from the Freundlich isotherm seem appropriate, the correlation coefficient (*R*^2^) is quite lower than that of the Langmuir isotherm (Table 6). Table 6 shows that the two adsorbents for the three kinds of dyes have an *n* value greater than one, indicating a more favorable adsorption process.^79,85^

**Table 6:** Isotherm fitting parameters of MO, CR and MB adsorption on adsorbents

Adsorbent	Dye	Langmuir				Freundlich			Temkin			Dubinin–Radushkevich				Scatchard		
		*Q*_max_ (mg g^−1^)	*K*_L_ (L mg^−1^)	*R*_L_	*R* ^2^	*n*	*K*_F_ (mg g^−1^ (L mg^−1^)^1/*n*^)	*R* ^2^	*B* (mg g^−1^)	*K*_T_ (L g^−1^)	*R* ^2^	*β* (mol^2^ kJ^−2^)	*Q*_m_ (mg g^−1^)	*E* (kJ mol^−1^)	*R* ^2^	*b* (L mg^−1^)	*Q*_s_ (mg g^−1^)	*R* ^2^
UiO-66(S)	MO	175.4	0.193	0.02	0.98	4.38	56.2665	0.77	29.88	0.657	0.96	1 × 10^−6^	145.4	707.1	0.92	0.21	170.8	0.93
	CR	144.9	0.206	0.02	0.99	7.21	71.3930	0.89	20.26	0.117	0.92	6 × 10^−6^	126.7	288.7	0.89	0.20	145.6	0.98
	MB	102.1	0.019	0.21	0.93	1.99	6.38400	0.87	25.06	0.159	0.84	4 × 10^−5^	67.84	111.8	0.66	0.01	135.3	0.63

Mesoporous UiO-66(U)	MO	188.6	0.815	0.01	0.95	5.35	81.7885	0.80	21.28	0.015	0.91	2 × 10^−7^	170.9	1581	0.95	0.86	184.9	0.87
	CR	147.1	1.152	0.01	0.97	5.67	71.8442	0.85	17.55	0.012	0.92	1 × 10^−7^	140.9	2236	0.86	0.96	153.4	0.91
	MB	107.5	0.028	0.15	0.96	2.67	10.1017	0.93	26.01	0.225	0.92	3 × 10^−6^	77.80	408.3	0.70	0.02	124.7	0.85

The Langmuir adsorption isotherm indicates that monolayer adsorption occurs on a homogeneous adsorbent surface and that all adsorption sites on the adsorbent surface have the same energy without adsorbate molecules migrating across the surface.^5,91^ The Langmuir adsorption isotherm model is given as (eqn (9)):9
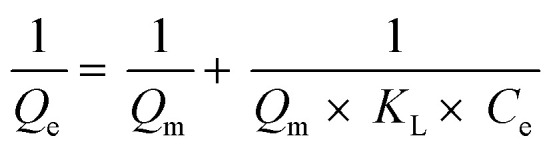
where *K*_L_ (L mg^−1^) indicates the Langmuir adsorption constant and *R*_L_ (dimensionless) describes the adsorption system as favorable or unfavorable.^77^

Data plots for the Langmuir isotherm models by mesoporous UiO-66(U) and UiO-66(S) for MO, CR, and MB at 30 °C are shown in Fig. 13(a). Table 6 displays the correlation coefficient values (*R*^2^) and *R*_L_ from the Langmuir isotherm model. Because the correlation coefficient value was larger than that of the Freundlich isotherm, the correlation coefficient values from the Langmuir isotherm model for the three kinds of dyes were better suited to representing the adsorption isotherm. Table 6 also shows that the *R*_L_ value is between 0 and 1, indicating that the adsorption is favorable.^4,12^ As shown in Table 3, the highest adsorption capacity value of mesoporous UiO-66(U) dyes was 188.7, 147.1, and 107.5 mg g^−1^, respectively. The comparison of the findings of this work with those of other studies (given in Table 9) demonstrates that mesoporous UiO-66(U) has the greatest adsorption performance for the adsorption of MO and CR (anionic) dyes. In contrast, the adsorption capacity for MB (cationic) dyes is slightly lower than that of other adsorbents. The most effective isotherm equations have been identified using error analysis techniques like correlation coefficient (*R*^2^), root-mean-square error (RMSE), and *χ*^2^ (see in ESI†). A smaller RMSE and *χ*^2^ value suggest a better model fit.

**Fig. 14:**
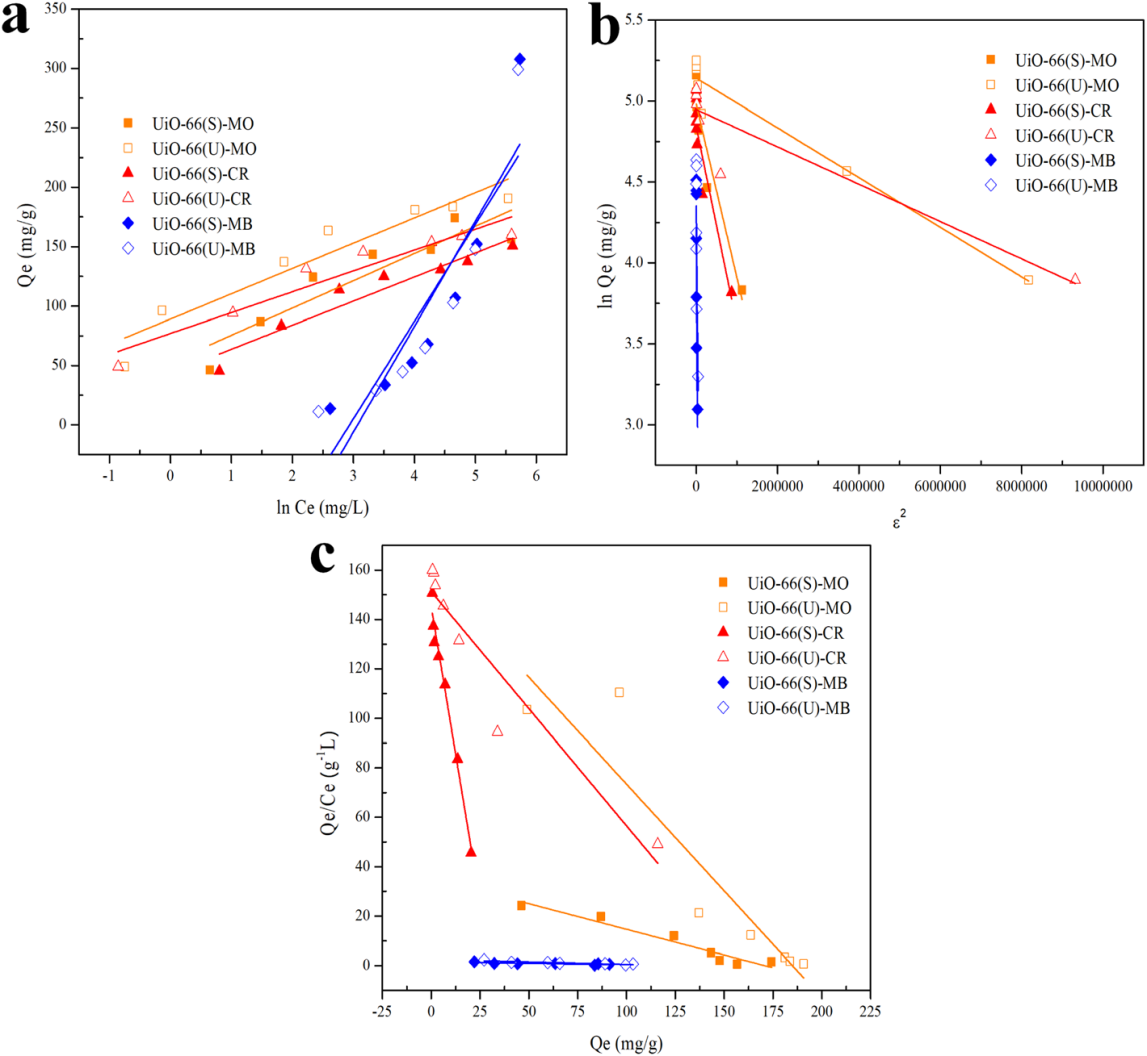
Model fits of (a) Temkin, (b) Dubinin–Radushkevich and (c) Scatchard isotherms with experimental adsorption data of MO, CR and MB on UiO-66(S) and mesoporous UiO-66(U).

Fig. 14 illustrates the plot of the Temkin, Dubinin, Radushkevich, and Scatchard adsorption isotherm models. Table 5 shows the adsorption isotherm parameter values obtained from MO, CR, and MB adsorption. Isotherm of adsorption Temkin suggested that the adsorbent–adsorbate interaction generates heat energy from adsorption.^92^ The deposited adsorbate might be dispersed uniformly across the layer throughout the adsorption process, reducing the heat of adsorption.^86^ The Temkin adsorption isotherm model (eqn (10)) is as follows:10
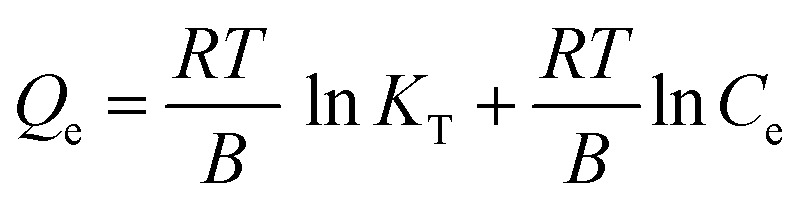
where *T* denotes the absolute temperature, *R* represents the gas constant (8.314 J mol^−1^ K^−1^), and *B* exhibits the Temkin isotherm constant. The value of parameter *B* in Table 5 indicates that adsorption was an exothermic process since it was greater than zero, resulting in heat release during adsorption.^93^

The Dubinin–Radushkevich isotherm model was applied to investigate the occurrence of physisorption with van der Waals forces. The Dubinin–Radushkevich isotherm equation could be employed to calculate the average adsorption-free energy. Physisorption occurs when the *E* value is below 8 kJ mol^−1^, but chemisorption occurs when the value is 8 < *E* < 16 kJ mol^−1^. Adsorption happens through the diffusion of chemical particles when the *E* value is more than 16 kJ mol^−1^.^12^ The Temkin parameters and the adsorption isotherm model can be represented as follows (eqn (11)–(13)):

11 ln *Q*_e_ = ln *Q*_m_ − *βε*^2^

12
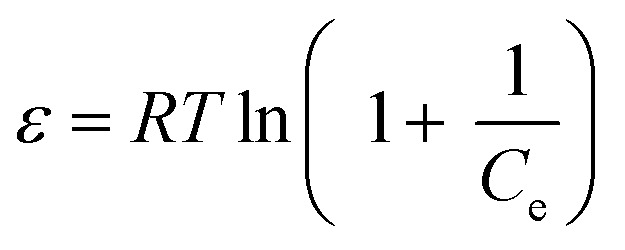
13
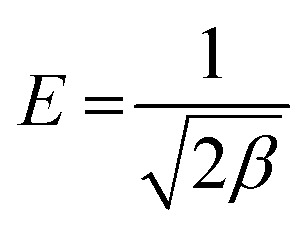
where *β* (mol^2^ J^−2^) a denotes the Dubinin–Radushkevich isotherm constant and *ε* exhibits the Polanyi potential, using eqn (12) to evaluate. Meanwhile, eqn (13) can be utilized to determine the value of *E*. The Dubinin–Radushkevich (D–R) isotherm can be used to calculate adsorbent porosity and adsorption energy.^94^

The Scatchard adsorption isotherm model was utilized to estimate the number of interactions that happened during the adsorption process between the adsorbate and the adsorbent. The Scatchard adsorption isotherm model (eqn (14)) is as follows:14
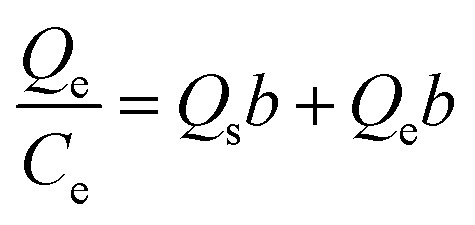


The Scatchard isotherm parameter is *Q*_s_ (mg g^−1^), while the Scatchard isotherm constant is *b* (L mg^−1^). The Scatchard adsorption isotherm was used to determine the number of dye-adsorption sites and their relative affinity.^95,96^

### Thermodynamic analysis

The temperature was used as the primary parameter to evaluate the energy changes that occur during the MO, MB, and CR adsorption on UiO-66. Thermodynamic studies were conducted at temperatures ranging from 30 to 50 °C. Van't Hoff plots were used to calculate thermodynamic parameters such as the change in Gibbs free energy, enthalpy, and entropy. This parameter could be used to explore either exothermic or endothermic spontaneous adsorption activities, and the degree of the disorder can be determined using the following equations (eqn (15) and (16)):^97,98^15
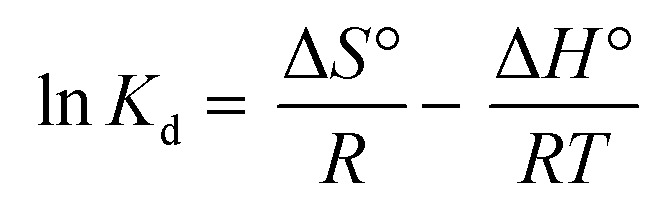

16 Δ*G*° = −*RT* ln *K*_d_

where Δ*S*° (J mol^−1^ K^−1^) represents the standard entropy change, *T*(K) symbolizes the adsorption temperature, and *R* (8.3145 J mol^−1^ K^−1^) denotes the gas constant. Meanwhile, Δ*H*° (kJ mol^−1^) indicates the enthalpy change, Δ*G*° (kJ mol^−1^) is the Gibbs free energy change, and *K*_d_ ((*Q*_e_/*C*_e_) 1000) denotes the kinetic energy change.^98^

**Fig. 15:**
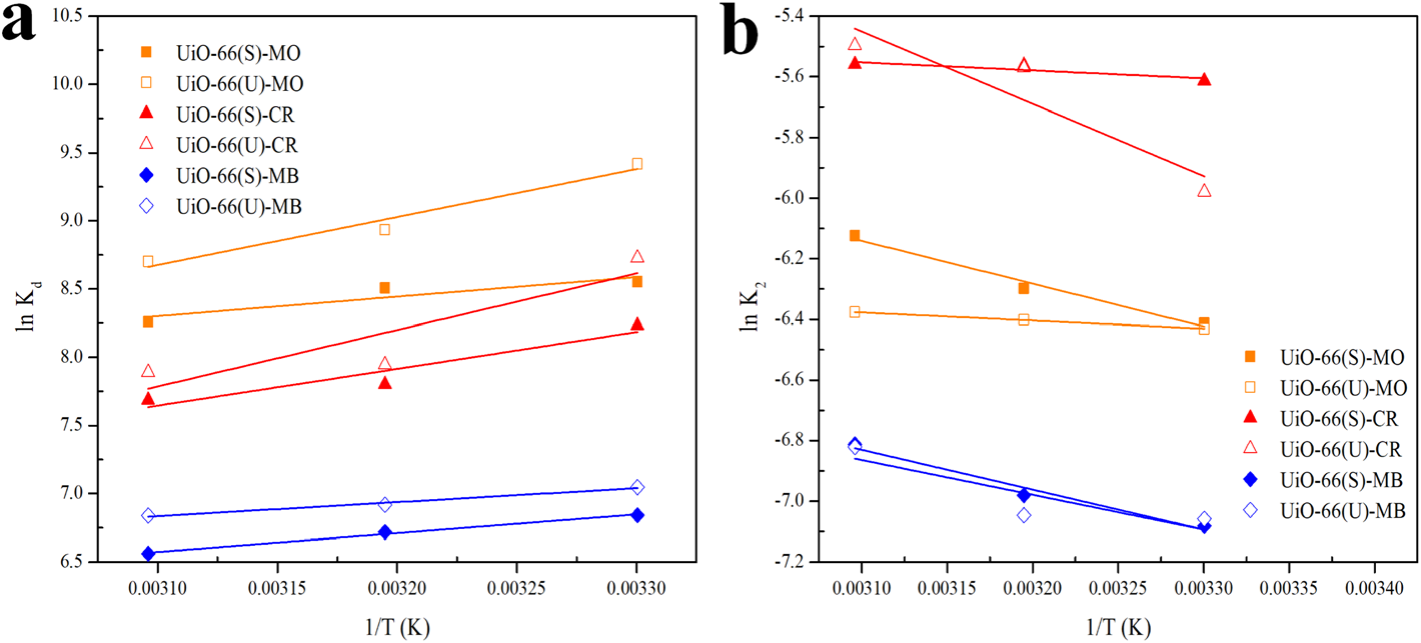
(a) Van't Hoff and (b) Arrhenius plot of MO, CR and MB adsorption onto UiO-66(S) and mesoporous UiO-66(U).

The slope and intercept of Van't Hoff linear plot shown in Fig. 15(a) and Table 7 could be used to determine the values of Δ*H*° and Δ*S*°. Because Δ*H*° was negative in this case, it indicated that the adsorption process did take place exothermically.^84^ In addition, since the value of Δ*H*° < 40 kJ mol^−1^, physisorption processes and endothermic adsorption reactions occurred in the adsorption process for the three dyes.^99^ Δ*H*° might also be negative due to weaker interactions between the adsorbate and the adsorbent.^100,101^

**Table 7:** Thermodynamic parameters for MO, CR and MB adsorption on adsorbents

Adsorbent	Dye	*E*_a_ (kJ mol^−1^)	*A* (g mg^−1^ min^−1^)	Δ*H*° (kJ mol^−1^)	Δ*S*° (J mol^−1^ K^−1^)	Δ*G*° (kJ mol^−1^)			*R* ^2^
						303 K	313 K	323 K	
UiO-66(S)	MO	11.736	0.17115	−11.849	32.283	−21.546	−22.138	−22.180	0.850
	CR	2.1904	0.00877	−22.400	5.8721	−20.742	−20.302	−20.642	0.910
	MB	10.938	0.06381	−11.381	19.374	−17.232	−17.483	−17.617	0.991

Mesoporous UiO-66(U)	MO	2.2151	0.00388	−29.278	18.632	−23.725	−23.249	−23.365	0.967
	CR	19.779	0.14602	−34.400	41.901	−21.985	−20.682	−21.185	0.816
	MB	9.5461	0.03671	−8.4836	30.527	−17.775	−18.002	−18.363	0.986

The standard Gibbs free energy change (Δ*G*°) was negative, suggesting that MO, CR, and MB adsorption to UiO-66 happened spontaneously and was thermodynamically highly favorable.^5,102^ A positive Δ*S*° result suggested that the adsorption process was becoming more random.^44^ It could be attributed to dye molecule adsorption on UiO-66 and the desorption of some water molecules from the adsorbent.^103^Table 8 summarizes the comparison of the adsorption thermodynamic characteristics of MO, CR, and MB on different kinds of adsorbents.

**Table 8:** Thermodynamic parameters for MO, CR and MB adsorption onto some adsorbents

Adsorbents	Dye	*T* (K)	Δ*G*° (kJ mol^−1^)	Δ*H*° (kJ mol^−1^)	Δ*S*° (J mol^−1^ K^−1^)	Ref.
Modulated Al_2_O_3_@UiO-66	MO	313	−2.303	−8.270	47.049	12
Sugar scum powder	MO	298	−14.63	−14.14	2.03	132
Wheat straw	MO	303	−9.51	−0.499	32	133
UiO-66(S)	MO	303	−21.546	−11.849	32.283	This study
Mesoporous UiO-66(U)	MO	303	−23.725	−29.278	18.632	This study
Porous activated kaolinite	MB	298.15	−2.10	37.0	128 900	134
Activated carbon from shrimp shell	MB	303.15	−9.997	18.86	95	102
ZIF-8	MB	303.15	−18.692	−35.916	−57.2	98
UiO-66(S)	MB	303	−17.232	−11.381	19.374	This study
Mesoporous UiO-66(U)	MB	303	−17.750	−8.483	30.527	This study
Cationic surfactant modified-biomass derived carbon	CR	298	−7.19	−43.86	−120	135
Zn_5_	CR	298	−32.11	17.50	166.16	136
Mn-UiO-66@GO-NH_2_	CR	303	−13.55	97.56	360.11	137
UiO-66(S)	CR	303	−20.742	−22.40	5.872	This study
Mesoporous UiO-66(U)	CR	303	−21.985	−34.40	41.90	This study

**Table 9:** Adsorption capacities summary of MO, CR and MB obtained in this study with various adsorbents reported in literature

Adsorbents	Dye	*Q*_max_ (mg g^−1^)	Ref.
Activated carbon from pinus	MO	91.9	138
MIL-53	MO	57	139
MCM-41/ZIF-67	MO	161.6	140
UiO-66-NH_2_-MnFe_2_O_4_–TiO_2_-TiNT	MO	164	141
UiO-66(S)	MO	175.4	This study
Mesoporous UiO-66(U)	MO	188.7	This study
γ-Fe_2_O_3_/Sep-NH_2_ composite	CR	126.4	142
Hollow ZnFe_2_O_4_	CR	16.58	143
α-Fe_2_O_3_ nanorods	CR	57.2	144
MnF_2_O_4_	CR	41.99	145
UiO-66(S)	CR	144.9	This study
Mesoporous UiO-66(U)	CR	147.1	This study
CoFe_2_O_4_/GO	MB	80.6	146
Fe_3_O_4_@SiO_2_@UiO-66-NH_2_	MB	116	107
MoS_2_–COOH@UiO-66-NH_2_	MB	253	147
Cu-BTC	MB	197	148
UiO-66(S)	MB	102	This study
Mesoporous UiO-66(U)	MB	107.5	This study

The following Arrhenius equation (eqn (17)) can be used to determine activation energy:17
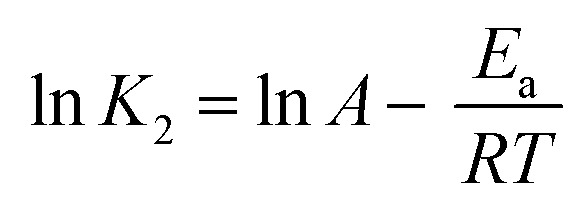
*E*_a_ (kJ mol^−1^) is the activation energy, *A* (g mg^−1^ min^−1^) is the Arrhenius constant, and *K*_2_ is the pseudo-second-order constant. Fig. 15(b) depicts the Arrhenius plot. Table 7 displays the values of *E*_a_, *A*, and the correlation coefficient (*R*^2^). According to the table, the activation energy in this research was quite low, showing that adsorption happened by physical interactions.^104,105^

### Influence of initial pH on dye adsorption

**Fig. 16:**
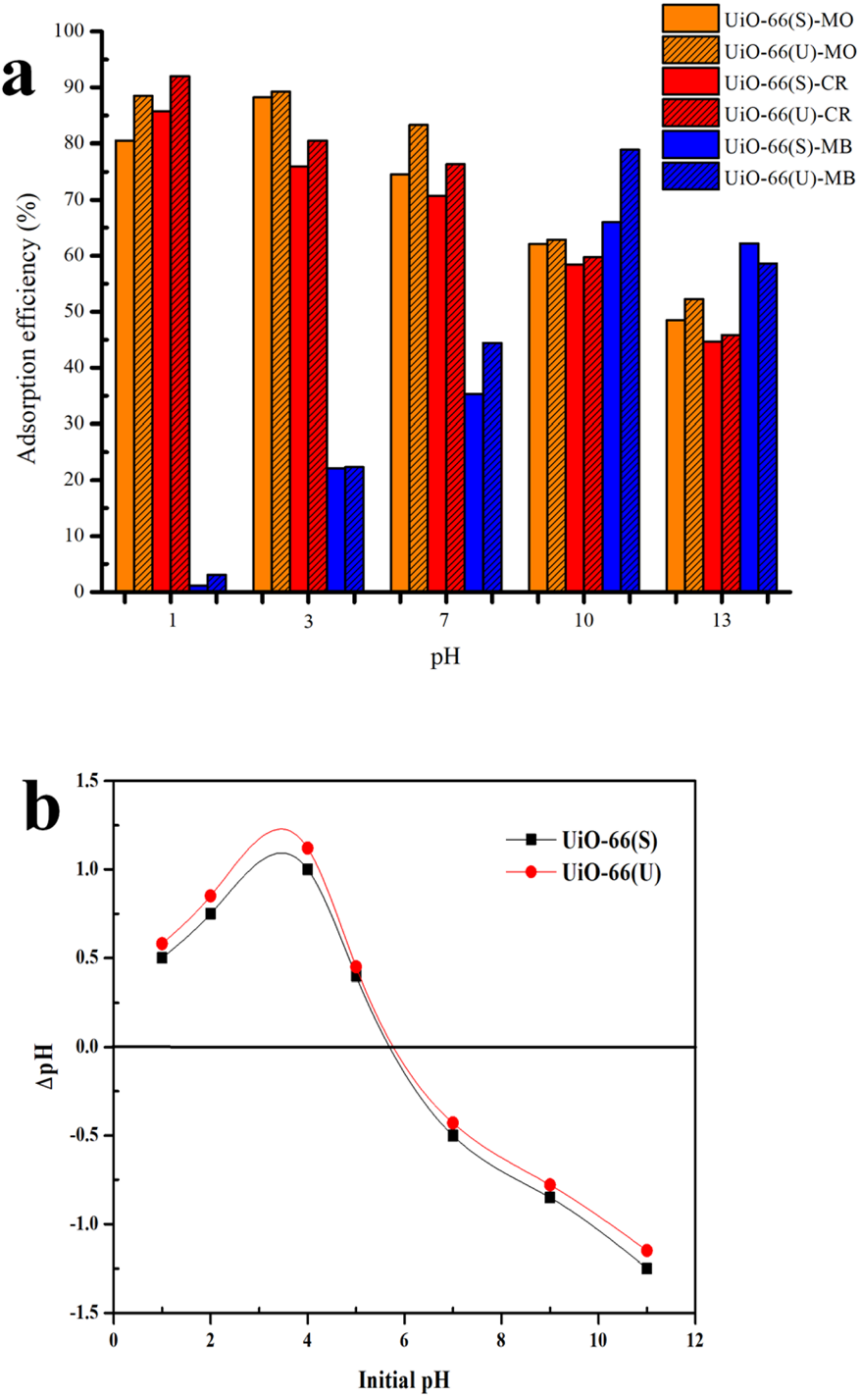
(a) Effect of initial pH on the adsorption MO, CR and MB on UiO-66(S) and mesoporous UiO- 66(U), (b) Plot to determine point of zero-charge (pHPZC) of UiO-66(S) and mesoporous UiO-66(U).

pH is a critical component in the adsorption process. The initial pH of the dye solution might influence the surface charge of the adsorbent and the dye molecule, which is strongly connected to their interaction. The effect of the dye solution's initial pH was studied in this work at 30 °C with a concentration of 100 ppm and a pH range of 1 to 13, and the results are shown in Fig. 16(a). The adsorption efficiency of anionic dyes (MO and CR) declined steadily with increasing pH, reaching its minimum at pH 13. Maximum MO adsorption efficiency was observed at a pH of 4, as the H^+^ ion concentration enhanced in an acidic environment and the adsorbent surface became more positively charged. Positively charged and anionic MO molecules exhibited strong electrostatic attraction at the adsorption site, producing a high adsorption efficiency for MO.^106^ The maximum CR adsorption efficiency was observed at pH 1, which might be attributed to the electrostatic interaction between the anionic dye and the highly positive charge of UiO-66. The adsorption of anionic dyes at alkaline pH, on the other hand, is still ongoing owing to the participation of additional adsorption processes such as the π–π stacking interaction between the aromatic structure of the dye molecule and the UiO-66 benzene ring, hydrogen bonding interactions, and physical adsorption and penetration of dye molecules into the UiO-66 pores.^4^ The cationic dye (MB) adsorption efficiency in both samples increased rapidly as the pH reached 10 (maximum) and subsequently dropped as the pH increased to 13. Because the adsorbent structure might be broken at an extremely high pH (1 and 13), the adsorption efficiency of the adsorbent could be diminished. Furthermore, according to Huang *et al.*,^107^ molecules are often present in dye solutions with a pH lower than the p*K*_a_ value when the pH is very severe. Therefore, there is no electrostatic interaction between the neutral dye and the positively charged adsorbent at pH values much below p*K*_a_.

On further review, this kind of trend can be explained by the fact that UiO-66 (U) was synthesized at 120 °C for 2 hours using a simple ultrasound-assisted method and used as an adsorbent to remove anionic dyes (MO and CR) and cationic dyes (MB) in the test. The pH_zpc_ test result in Fig. 16(b) shows that the surface charge of mesoporous UiO-66(U) and UiO-66(S) decreases with increasing pH, indicating the presence of negative surface charge. In this work, the pH_zpc_ value or isoelectric point of UiO-66 was 5.8, suggesting that the adsorbent surface was positively charged at pH < 5.8 and negatively charged at pH > 5.8.^46^ In previous studies, the pH_zpc_ UiO-66 value reached 5.^44^ In contrast, the pH_zpc_ of UiO-66 was 6.4 in another investigation.^108^ Meanwhile, it is known that the dissociation constant (p*K*_a_) of methyl orange, Congo red, and methylene blue are 3.4, 4.1, and 3.8.^109–111^ At pH < p*K*_a_, the solute tends to contain more protons, and the adsorbent's surface becomes more positively charged. This phenomenon arises due to electrostatic repulsion between the surface of the adsorbent and the dye, resulting in a decrease in adsorption. Conversely, when the pH value is higher than the p*K*_a_ of the dye but lower than pH_zpc_ UiO-66, or 2< pH < pH_zpc_ UiO-66 (5.8), the MO and CR molecules will deprotonate into anionic forms when the surface of UiO-66 is positively charged because the pH < pH_zpc_ resulting in electrostatic attraction leading to a considerable increase in adsorption.^110^

### Influence of ionic strength on adsorption capacity

**Fig. 17:**
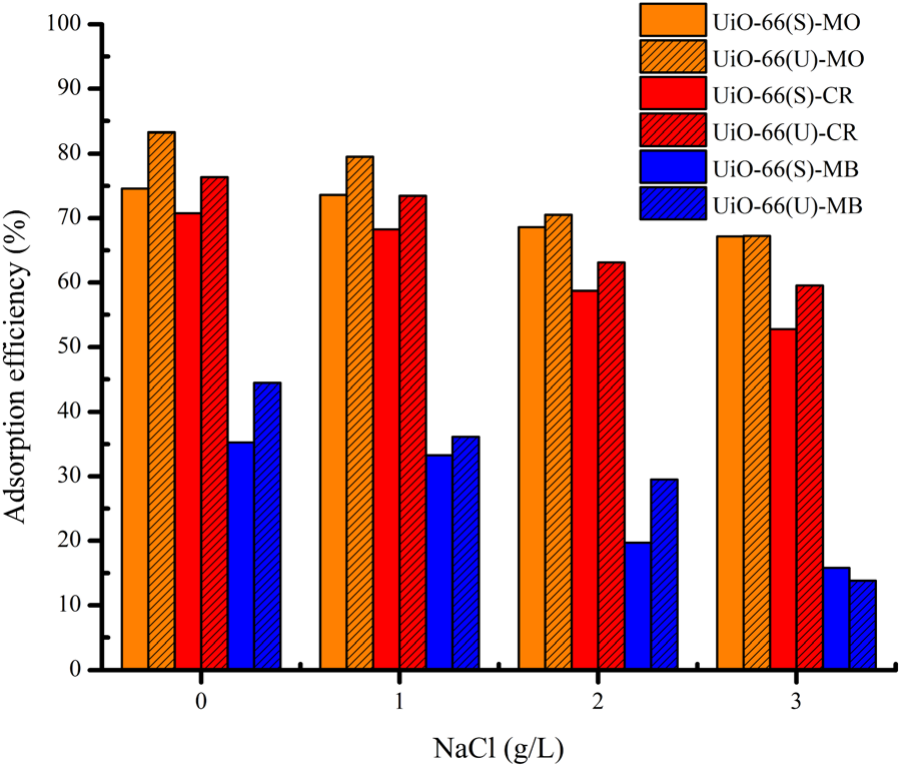
Effect of ionic strength on the adsorption MO, CR and MB on UiO-66(S) and mesoporous UiO-66(U).

Other than dyes, specific salt ions are prevalent in wastewater; therefore, salt concentration (ionic strength) might impact adsorption efficiency. At varied NaCl concentrations, the impact of ionic strength on the adsorption efficiency of the dyes in the two samples was investigated. Theoretically, when the ionic strength increases, the adsorption capacity decreases owing to electrostatic interactions between the dye molecules and the adsorbent, which have opposing charges. Conversely, the adsorption capacity will increase if there is no electrostatic interaction between the dye and the adsorbent due to the dye dissociation.^5^ The trend of anionic and cationic dye adsorption efficiency in UiO-66 declined as the NaCl content changed from 0 to 3 g L^−1^, as shown in Fig. 17. It might be because of the presence of chlorine ions (Cl), which can diminish the electrostatic interaction between dye molecules and adsorbent surfaces.^4^

### Selective adsorption behavior of mesoporous UiO-66

**Fig. 18:**
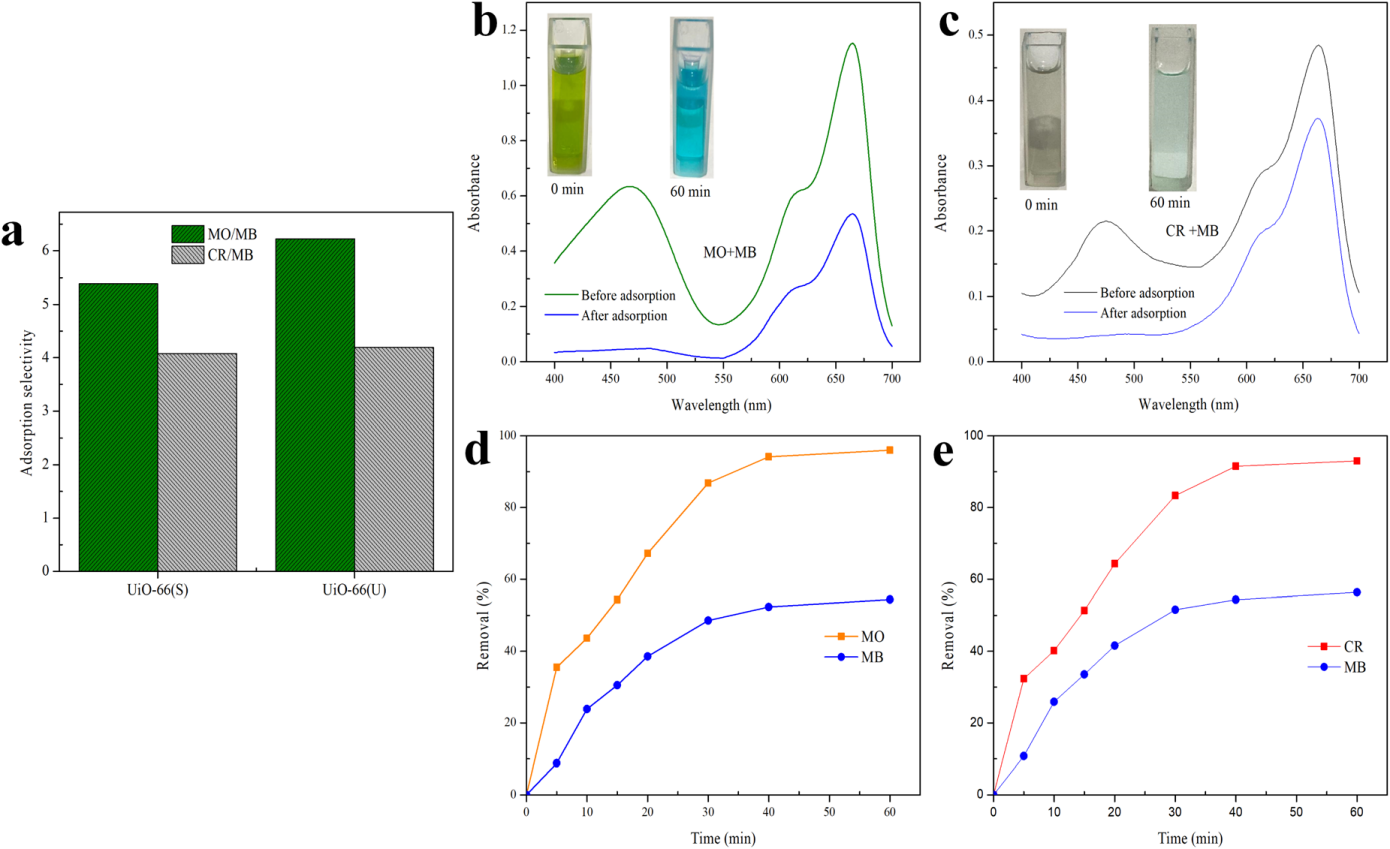
(a) The adsorption selectivity of MB over MO and MB over CR by mesoporous UiO-66(U) in single solution; UV-vis spectra and adsorption images of different mixtures (b) MO/MB and (c) CR/MB before and after adsorption onto the mesoporous UiO-66(U); adsorption removal to (d) MO/MB mixture and (e) CR/MB mixture as a function of time by mesoporous UiO-66(U).

To further investigate the adsorption selectivity of UiO-66 dye, two anionic dyes (MO and CR) and one cationic dye (MB) were used to evaluate the adsorbent's selectivity. According to eqn (3), the expected selectivity of MO over MB for UiO-66(S) and mesoporous UiO-66(U) was approximately 5.39 and 6.23, respectively, while the selectivity of CR over MB was around 4.08 and 4.19 for UiO-66(S) and mesoporous UiO-66(U) (Fig. 18(a)), respectively. The adsorption selectivity of MO/MB was much greater than that of CR/MB, which might be attributed to the larger geometric configuration of CR, which makes it more difficult to diffuse and reach the inner pores.^81,112^ Meanwhile, the increased pore size of mesoporous UiO-66(U) could effectively aid in the diffusion of MO and CR dyes.

In the following adsorption selectivity study, employing a dye mixture of 10 mg L^−1^ each, mesoporous UiO-66(U) demonstrated a very high MO/MB selectivity. Using a mixture of MO/MB and CR/MB dyes, the adsorption selectivity properties of mesoporous UiO-66(U) for MO and CR were examined. Fig. 18(b) and (c) exhibit UV-vis spectra and mixed dye photographs before and after adsorption. Mesoporous UiO-66(U) showed the most significant adsorption impact on the anionic dyes from the two mixed dyes. The UV-vis spectra of the MO/MB mixed dye before adsorption contains two prominent distinctive peaks at 464 nm (MO) and 665 nm (MB), as shown in Fig. 18(b). The distinctive MO peak was significantly attenuated after adsorption by mesoporous UiO-66(U), whereas the MB peak showed a less substantial drop. The color of the combined hue solution changed from green to blue (inset in Fig. 18(b)). This color shift after adsorption shows that the adsorbent has absorbed most of the MO (anionic) and the residual MB (cationic) in the solution.^13,77,113^ As shown in Fig. 18(c), the hue of the dye solution changed from blackish gray to light blue following the adsorption of both CR and MB mixed dyes by mesoporous UiO-66(U), and the distinctive peaks at 498 and 665 nm for CR and MB diminished. It should be noted that the CR peak decreased more significantly than the MB peak, indicating that mesoporous UiO-66(U) could effectively and fast adsorb cationic and anionic dyes for 60 minutes. However the anionic dye adsorption capacity was more remarkable.^4,13^

As shown in Fig. 18(d) and (e), the percentage of mesoporous UiO-66(U) removal increased with contact time and reached equilibrium in 60 minutes. Furthermore, according to Fig. 18(d), mesoporous UiO-66(U) exhibits removal percentages in the MO/MB combination for MO and MB of 96 and 54% CR after 60 minutes, respectively. Meanwhile, Fig. 18(e) reveals that the percentages of CR and MB removal are 93 and 56%, respectively. As evidenced by adsorption data, MO or CR molecules are more likely than MB molecules to bond with positively charged adsorbent sites.^4,13,44,113^ These findings are consistent with estimates of adsorption selectivity obtained from single adsorption data.

### Possible dye adsorption mechanisms

**Fig. 19:**
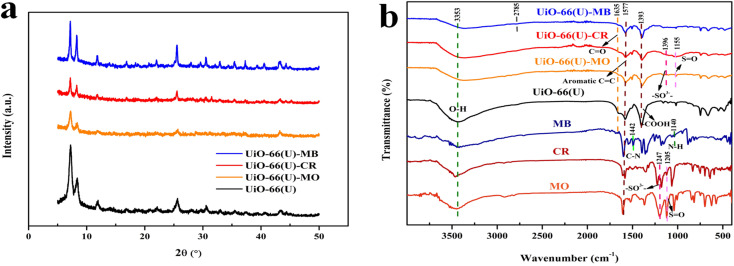
(a) XRD patterns for reused mesoporous UiO-66(U) after 4 adsorption–desorption cycles of dyes and (b) FTIR spectra of pure MO, MB, CR and before and after adsorption on mesoporous UiO-66(U).

The adsorption mechanisms of MO, CR, and MB on mesoporous UiO-66(U) can be described by several adsorbent–adsorbate interactions. Many adsorption mechanisms have been proposed to elucidate the adsorption of various dyes.^58,77^ In order to better comprehend the adsorption mechanism in both single and mixed dye systems, pH_zpc_, XRD, and FTIR were used. The XRD pattern of mesoporous UiO-66(U) before and after the third application is shown in Fig. 19(a), and it can be seen that the crystal structure of mesoporous UiO-66(U) maintains since there is no change in the peak position of mesoporous UiO-66(U) after the third usage.^58^ However, following the adsorption of MO and CR, the peak intensity of mesoporous UiO-66(U) decreased. It might be due to more MO and CR molecules interacting with the adsorbent's active site.^114^ Meanwhile, there was a slight drop in XRD mesoporous UiO-66(U) after the third usage for MB adsorption because MB is a cationic dye. On positively charged adsorbent surfaces, adsorption is typically weaker.

Numerous studies have reported that surface area is a crucial aspect that influences the adsorption process, and one of them is pore filling or physisorption.^115–117^ Most dye molecules are promptly adsorbed into the UiO-66 micropore channels and crystal surface. However, the movement of dye molecules is limited because of the tiny pores, large specific surface area, and pore volume. The inclusion of some mesoporous holes enhances the molecular mass transfer rate, allowing more toluene to enter the material, and considerably increases the adsorption capacity of mesoporous UiO-66(U).^20^Table 2 shows that mesoporous UiO-66(U) has a lower outer surface area than UiO-66(S) but a bigger pore size and greater pore volume. This could be one of the reasons why mesoporous UiO-66(U) can more efficiently adsorb all three kinds of dyes than UiO-66(S). In addition, mesoporosity significantly impacts the adsorption of organic dyes.^12^ Avogadro software was used to investigate the dye dimensions, which comprised 1.60 × 0.42 × 0.26 nm (MO), 1.38 × 0.43 × 0.65 nm (MB), and 2.50 × 0.78 nm (CR) (Fig. 19). The pore size of the mesoporous UiO-66(U) material is greater than the molecular size of the three dyes, allowing dye molecule diffusion to be more accessible and supporting a high adsorption capacity value. However, pore size and surface area are not critical considerations in determining adsorption capacity since mesoporous UiO-66(U) has a lower MB adsorption capacity.^115^

Electrostatic interactions take place in one dye adsorption system, which is controlled by the pH of the solution.^11,77^ The pH of the solution influences the state of the dye molecules as well as the surface charge of the adsorbent, both of which are critical to the adsorption process. The adsorption capacities of MO and CR steadily declined with increasing pH, as illustrated in Fig. 15(a), but the adsorption capacity of MB increased. The isoelectric point (pH_zpc_) study of the mesoporous UiO-66(U) surface was performed at pH 5.8, as shown in Fig. 15(b). As a result of the repulsion between MB and the positively charged mesoporous UiO-66(U) surface, the adsorption capacity of MB may be meager below pH 7.^113,115^ Electrostatic interactions will attract negatively charged anionic dye molecules to positively charged mesoporous UiO-66(U) in an acidic or neutral environment.^11^ If reviewed further, the dissociation constant of benzene dicarboxylic acid (p*K*_a_) is 3.51. It is important to remember that the constant acidity value applies to water, not DMF.^13,118^ Another adsorption mechanism that may take place is ion exchange between the benzene dicarboxylic acid ligand and the anion of the dye.^119^ The active site of the dye anion can interact with the metal *via* ion exchange, thus making such anions like ligands. To further understand the adsorption process, the FTIR spectra of the three dyes before and after adsorption on mesoporous UiO-66(U) were compared. Adsorption of MO or CR onto mesoporous UiO-66(U) caused several alterations in the absorption peaks (Fig. 19(b)). According to Fig. 19(b), MO and CR exhibit stretching vibrations S=O and C–S at wave numbers 1200–1250 cm^−1^ and 620 cm^−1^, respectively.^120^ Following the adsorption process, there was an interaction between the anionic dye's sulfonate group and the metal center mesoporous UiO-66(U), as demonstrated by the new absorption peak at wave numbers ranging from 1155–1396 cm^−1^.^121,122^ Meanwhile, the absorption peak of the stretching vibration of the carboxyl group of mesoporous UiO-66(U) diminished after MB adsorption, which might be attributed to the formation of hydrogen bonds after MB adsorption.^113,123^ Additionally, a shift in the absorption peak at a wave number of 1577 cm^−1^ is visible in Fig. 19(b), which represents the vibration of the aromatic ring and is attributed to the π–π stacking interaction between the adsorbent molecule and the dye.^11,113,124^ Another peak change in absorption occurred at 3433 cm^−1^, shifting to 3353–3372 cm^−1^, suggesting that the hydroxyl group could be significant in adsorption. Furthermore, there was an increase in intensity and a change in the absorption peaks at 2785 and 1400 cm^−1^, which moved to 1393 cm^−1^, associated with the stretching vibrations of the C–H group and the carboxyl group, which hydrogen bonds between the adsorbent and MB might cause.^5,123^ Further examination reveals a change in the absorption peak at 1635 cm^−1^ caused by the interaction of the dye molecule's carbonyl group with the surface charge of the adsorbent group through hydrogen bonding, as seen in Fig. 19.^5^

**Fig. 20:**
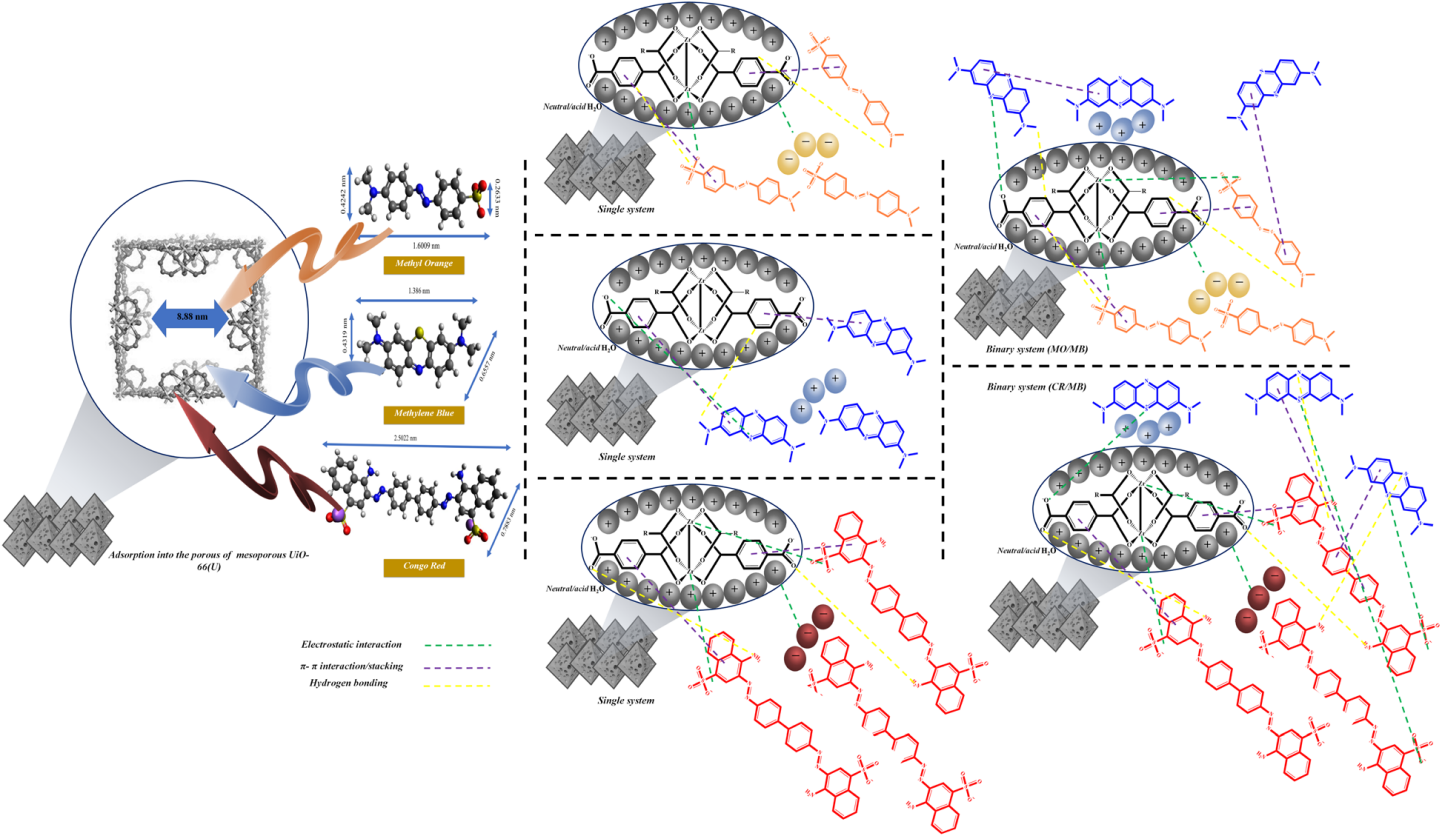
The possible mechanism for the adsorption of MO, CR and MB onto mesoporous UiO-66(U).

The difference in adsorption capacity in the binary system might be due to competition between the two dye molecules at the adsorption site.^5,77^ Although it has strong selectivity for anionic dyes and a positive surface charge suggests an interaction between the two dye molecules in a binary system, there is evidence that mesoporous UiO-66(U) can adsorb MB in the MO/MB and CR/MB mixtures. Previous research has shown that adding a second dye molecule in a binary system could boost the adsorption capacity of the first dye molecule owing to the existence of functional groups in the second dye molecule that can bind to the first dye molecule, such as *via* π–π interactions.^125^ According to Li *et al.*,^126^ a push–pull mechanism between two dye molecules in a binary system might enhance the adsorption process. For example, cationic dye molecules can encourage anionic dimerization, and anionic dimers can attract cationic molecules together to be adsorbed on the adsorbent. Dye molecules can self-associate to form dimers through π–π stacking interactions between aromatic rings.^127,128^ As depicted in Fig. 20, MO or CR dimers with a negative charge at either end might attract MB molecules, or interacting with the zirconium cluster permits MB molecules to be adsorbed.

### Regeneration

**Fig. 21:**
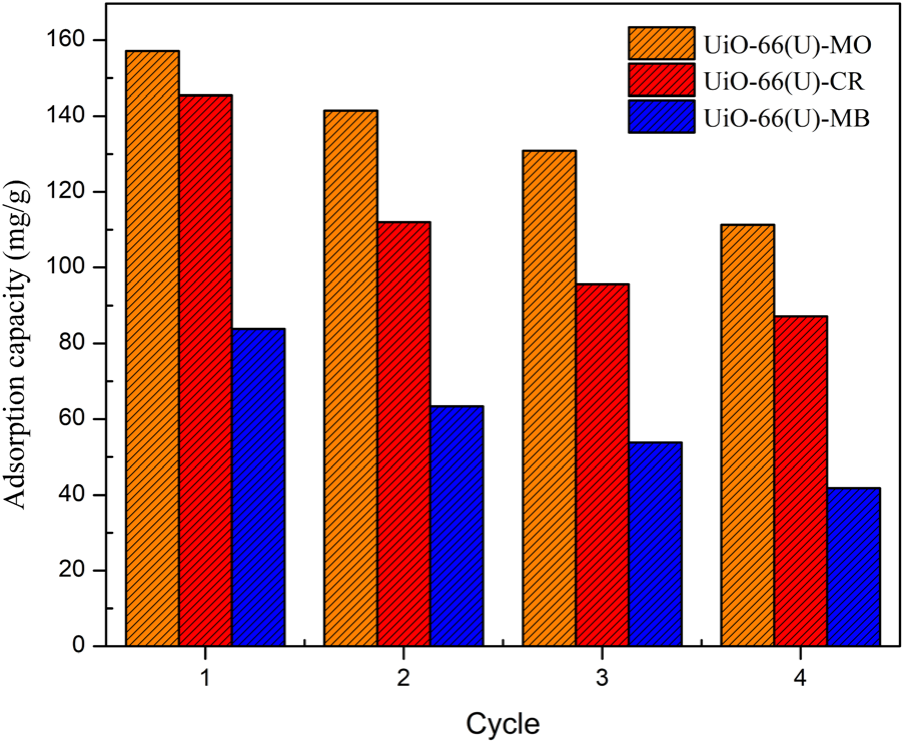
Effect of recycle times on the adsorption capacities of MO, CR and MB on mesoporous UiO-66(U).

In industrial applications, regeneration and reuse of adsorbents are often crucial. Utilizing the adsorbate desorption process with 0.01 M HCl solution and demineralized water as the eluent, the reuse of mesoporous UiO-66(U) for the three types of dyes for up to four cycles was investigated. Mesoporous UiO-66(U) dye samples were dispersed in HCl solution for 90 minutes before being rinsed with methanol and ethanol several times.^129,130^ It is then dried at 100 °C and reused for subsequent adsorption procedures. Fig. 21 demonstrates that the adsorption capacity of the three dyes did not decrease significantly after regeneration. This modest drop was due to trapped dye molecules that might have covered the adsorbent's active site.^4,131^ The results of this regeneration indicate that mesoporous UiO-66(U) is a highly efficient and effective adsorbent, even after several utilizations.

## Conclusions

Mesoporous UiO-66(U) was synthesized using ultrasonic irradiation without organic or inorganic templates. This method provides a fast, effective, and template-free route for synthesizing mesoporous UiO-66(U). XRD and FTIR spectra confirmed the mesoporous phase and structure of UiO-66(U). The results of the SEM analysis showed that the size of the mesoporous particles of UiO-66(U) and UiO-66(S) was 150 nm. The pore diameters of the mesoporous materials UiO-66(U) and UiO-66(S) are 8.88 and 3.39 nm, respectively. Ultrasonic irradiation plays an essential role in the emergence of mesopores during the reaction process. Based on the results obtained here, it can be concluded that high energy and highly irregular collapsing bubbles lead to the formation of local hot spots, microjets, and free radicals, resulting in the production of mesoporous UiO-66(U) particles with larger pores and irregularities. More than 97% of energy savings were achieved in the sonochemical-solvent heat mixing compared to conventional solvothermal methods. Compared to UiO-66(S) synthesized by conventional solvothermal procedures, the ultrasonic process yielded significantly superior material properties and adsorption performance. The anionic and cationic dye adsorption processes are remarkably consistent with the pseudo-second-order model and the Langmuir isotherm model, both of which have spontaneous and exothermic characteristics. The adsorption performance trial revealed that mesoporous UiO-66(U) had more excellent MO (188.68 mg g^−1^), CR (147.05 mg g^−1^), and MB (107.52 mg g^−1^) adsorption capacities than UiO-66(S). The adsorption performance of the mixed dyes further demonstrated that mesoporous UiO-66(U) could concurrently adsorb anionic and cationic dyes, but anionic dyes were significantly more selectively adsorbed. One limitation of this work is that the surface area of mesoporous UiO-66(U) synthesized by the ultrasound method is small and therefore needs further development. In addition, further adsorption studies must be conducted in binary and ternary systems containing various contaminants, such as pharmaceutical waste, heavy metals, and other organic pollutants.

## Conflicts of interest

“There are no conflicts to declare”.

## Supplementary Material

RA-013-D2RA06947D-s001
