# Supplementary material for: Selective adsorption of anionic and cationic dyes on mesoporous UiO-66 synthesized using a template-free sonochemistry method: kinetic, isotherm and thermodynamic studies
Source: RSC Adv. 2023 Apr 20;13(18):12320–43. doi: 10.1039/d2ra06947d (PMC10116191; doi:10.1039/d2ra06947d)
Supplement: RA-013-D2RA06947D-s001 [file RA-013-D2RA06947D-s001.pdf]

Electronic Supplementary Material (ESI) for RSC Advances.
This journal is © The Royal Society of Chemistry 2023

*Supplemantary Materials*

# Selective adsorption of anionic and cationic dyes on mesoporous UiO-66 synthesized using a template-free sonochemistry method: Kinetic, isotherm and thermodynamic studies

**Alvin Romadhoni Putra Hidayat[a], Liyana Labiba Zulfa[a], Alvin Rahmad Widyanto[a], Romario Abdullah[a], Yuly Kusumawati[a], Ratna Ediati[a,*]**

[a]*Department of Chemistry, Faculty of Science and Data Analytics, Institut Teknologi Sepuluh Nopember, Jl. Arif Rahman Hakim, Kampus ITS Keputih-Sukolilo, Surabaya 60111, Indonesia*

** Corresponding Author. E-mail: rediati@chem.its.ac.id;*
*Telp: +62-31-5943353, Fax: +62-31-592831*

**Synthesis Yield of UiO-66**

The stoichiometric chemical reaction of $ZrCl_4$ with 1.4-benzenedicarboxylic acid ($C_8H_6O_4$) in the UiO-66 ($Zr_6O_4(OH)_4(C_8H_4O_4)_6$) synthesis is

$$6ZrCl_4 + 6C_8H_4O_4 + 8H_2O \rightarrow Zr_6O_4(OH)_4(C_8H_4O_4)_6 + 24HCl$$

The synthesis yield of the UiO-66 was calculated using following equation.

$$Yied(\%) = \frac{m_{exp}}{m_{theo}} x100\%$$

where $m_{exp}$ is the mass of UiO-66 produced (g), and $m_{theo}$ is the theoretical mass of UiO-66 based on stoichiometry (g), where $m_{exp}$ of UiO-66 is 1.33 g, and $m_{theo}$ of UiO-66(S) and Mesoporous UiO-66(U) respectively are 1.16 g and 1.29 g. Whereas the $Yield(\%)$ for UiO-66(S) and Mesoporous UiO-66(U) respectively are 87% and 97%.

**Table S1**. The characteristic peaks of XRD diffractogram of UiO-66

| 2θ (°) | References |
|---|---|
| 7.38, 8.56, 25.67 | [1] |
| 7.4, 8.6, 25.7 | [2] |
| 7.1, 8.2, 25.5 | [3] |
| 7.18, 8.35, 25.5 | [4] |
| 7.25, 8.39, 25.63 | This study (solvothermal) |
| 7.27, 8.47, 25.62 | This study (ultrasound) |

**Table S2.** Weight Distribution of the Elements Within the Composition

| Material | Elements | percentage (%) |
|---|---|---|
| UiO-66(S) | Zr | 15.61 |
| | O | 19.94 |
| | C | 64.44 |
| Mesoporous UiO-66(U) | Zr | 37.30 |
| | O | 19.30 |
| | C | 43.30 |

**Energy calculation for synthesis**

One of the main advantages of using ultrasound as the synthesis route is that it consumes less energy than conventional solvothermal. By the conventional solvothermal method, UiO-66 was synthesized by dissolving $ZrCl_4$ and $H_2BDC$ for 15 minutes in 45 mL DMF solution with a mole ratio of 1:1. The two solutions were mixed and stirred with magnetic stirring at 350 rpm for 30 minutes. After stirring for 30 minutes, the mixture was heated in an oven for 24 hours at a temperature of 120 °C. Then the material was washed, centrifuged, and dried at 90 °C for 3 hours. The energy consumption in both synthesis methods was calculated as follows:

**Conventional solvothermal method**

• Total power consumed during stirring in second = 150 J/s

• For a total of 15 minutes of stirring for metal precursors, total energy consumed = 150 (J/s) × 0.25 (h) × 3600 (s) = 135 kJ

• For a total of 15 minutes of stirring for the ligand precursor, the total energy consumed = 150 (J/s) × 0.25 (h) × 3600 (s) = 135 kJ

• For a total of 30 minutes of stirring to mix the metal precursor solution and the ligand, the total energy consumed = 150 (J/s) × 0.55 (h) × 3600 (s) = 270 kJ

• Total amount of material processed = 1.0487 g $ZrCl_4$ + 0.748 g $H_2BDC$ + 90 mL DMF = 86.757 g

• Total power consumed during oven or solvothermal heating in seconds = 800 J/s

• For a total of 24 hours of stirring for heating by oven or solvothermal, the total energy consumed = 800 (J/s) × 24 (h) × 3600 (s) = 69120 kJ

• Total energy consumed during synthesis per unit amount of material = (69660/86.757) = 802.93 kJ/g

**Ultrasonic-solvent heat mixing method**

• Total power consumed during stirring in second = 150 J/s

• For a total of 45 minutes of stirring for the precursor = 540 kJ

• Total amount of material processed = 86,757 g

• Total power consumed during ultrasonic irradiation (ultrasonic probe) in seconds = 120 J/s

• For a total of 24 hours during heating and ultrasonic irradiation, the total energy consumed = (120 (J/s) × 2 (h) × 3600 (s)) + (150 (J/s) × 2 (h) × 3600 (s)) = 1944 kJ

• Total energy consumed during synthesis per unit amount of material = (1944/86,757) = 22.41 kJ/g

Thus, the ultrasonic-solvent heat mixing approach saves 97.21% more energy than the standard solvothermal method, obtained from (Total energy conventional/Total energy ultrasonic)/Total energy conventional x 100%.

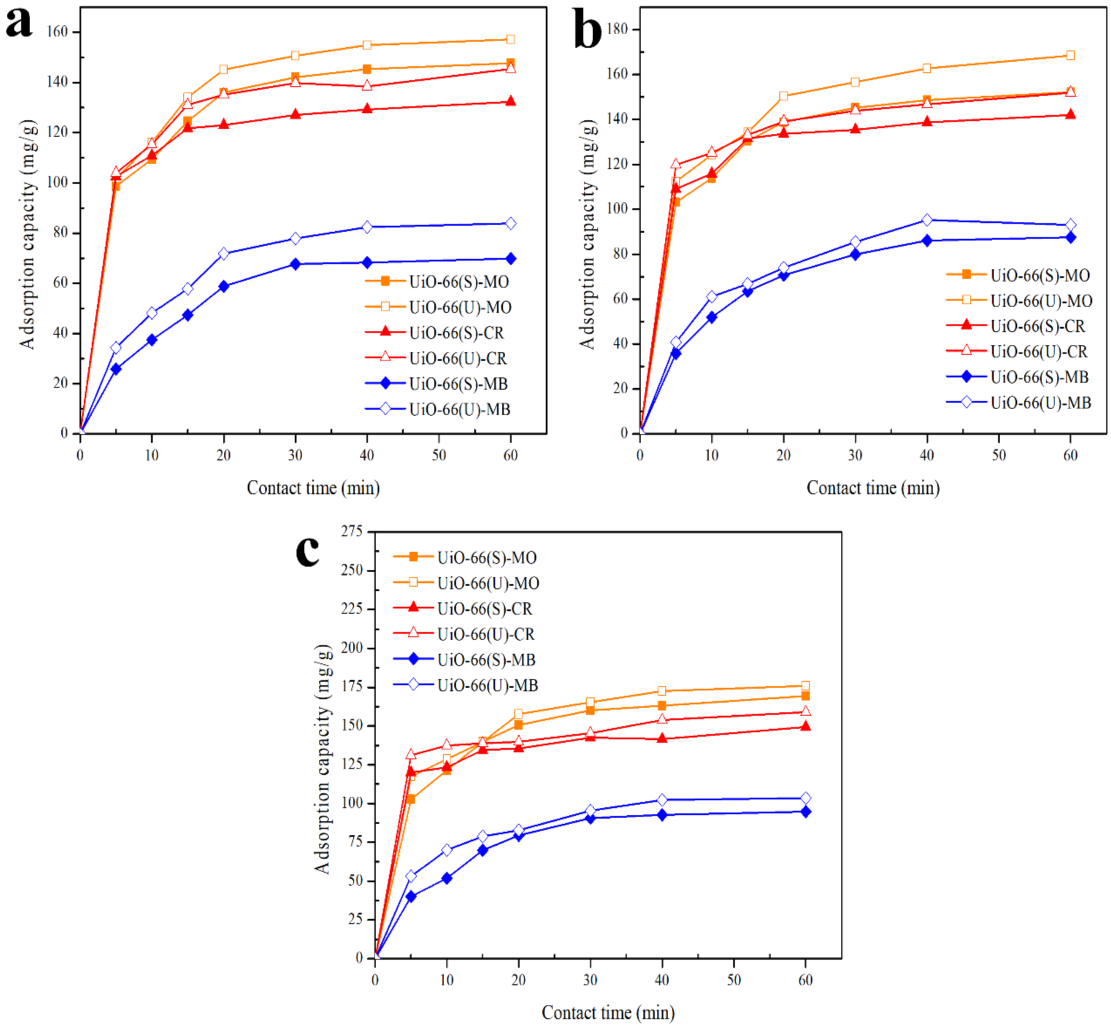


**Fig. S1.** Effect of contact time on adsorption of MO, CR and MB by UiO-66(S) and Mesoporous UiO-66(U) at 30 °C and initial concentration of (a) 100, (b) 150, (c) 200 mg/L.

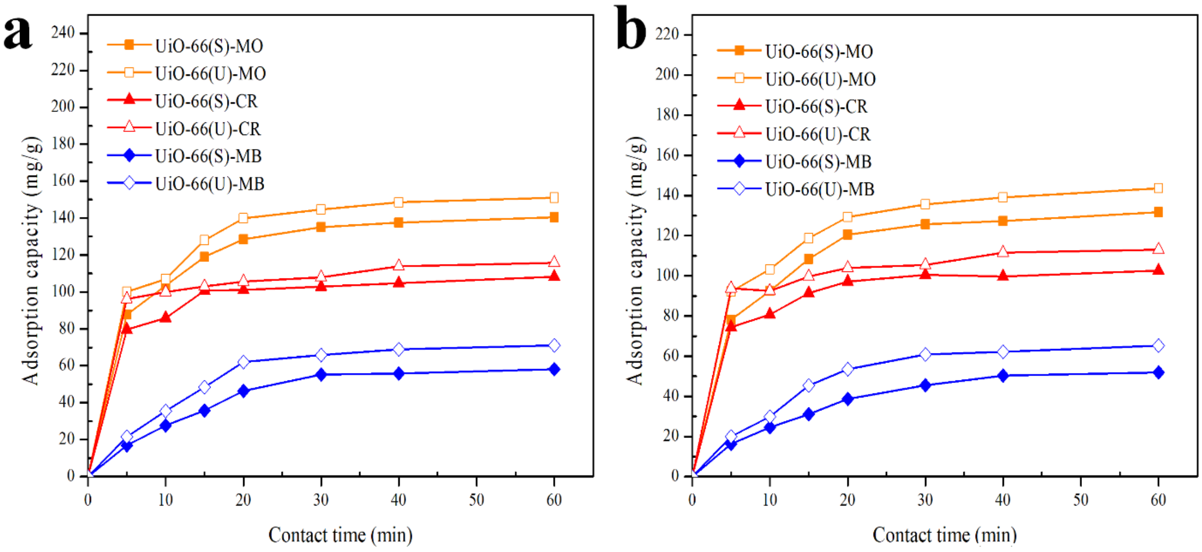


**Fig. S2.** Effect of contact time on adsorption of MO, CR and MB by UiO-66(S) and Mesoporous UiO-66(U) at (a) 40 and (b) 50 °C.

The following parameters might represent the applicability of the isotherm model when applied to experimental data: $R^2$ after linear regression, rootmean-square error (RMSE), $\chi^2$ (chi-square).[5] Smaller RMSE and $\chi^2$ (chi-square) values imply a greater level of model fit. The RMSE and $\chi^2$ (chi-square) equations are represented as **Eqs.(1)** and **(2)** as follows.[6–8]:

$$RMSE = \sqrt{\frac{1}{n-2}\sum_{i=1}^{n}(Q_{exp} - Q_{cal})^2} \quad (1)$$

$$\chi^2 = \sum_{i=1}^{n}\frac{(Q_{exp} - Q_{cal})^2}{Q_{cal}} \quad (2)$$

where $Q_{exp}$ (mg/g) is the experimental adsorption capacity, $Q_{cal}$ (mg/g) is the adsorption capacity predicted using the isotherm model, respectively, and n is the number of experimental samples.

| Adsorbents | Langmuir | | | Freundlich | | |
|---|---|---|---|---|---|---|
| | $R^2$ | RMSE | $\chi^2$ | $R^2$ | RMSE | $\chi^2$ |
| UiO-66(S)-MO | 0.9884 | 0.00075 | 0.000254 | 0.7721 | 0.2717 | 0.0648 |
| UiO-66(U)-MO | 0.9527 | 0.00146 | 0.000714 | 0.8048 | 0.2679 | 0.0631 |
| UiO-66(S)-Cr | 0.9974 | 0.00034 | 0.000057 | 0.8936 | 0.2905 | 0.0768 |
| UiO-66(U)-Cr | 0.9764 | 0.00097 | 0.000441 | 0.8535 | 0.2028 | 0.0358 |
| UiO-66(S)-MB | 0.9353 | 0.00399 | 0.003032 | 0.8707 | 0.2416 | 0.0547 |
| UiO-66(U)-MB | 0.9631 | 0.00233 | 0.001211 | 0.9266 | 0.1457 | 0.0246 |
